# Cluster randomised trials with a binary outcome and a small number of clusters: comparison of individual and cluster level analysis method

**DOI:** 10.1186/s12874-022-01699-2

**Published:** 2022-08-12

**Authors:** Jennifer A. Thompson, Clemence Leyrat, Katherine L. Fielding, Richard J. Hayes

**Affiliations:** 1 Department of Infectious Disease, London School of Hygiene & Tropical Medicine, London, UK; 2 Department of Medical Statistics, London School of Hygiene & Tropical Medicine, London, UK

**Keywords:** Cluster-level analysis, Cluster level analysis, Generalised linear mixed model, Generalised estimating equations, Comparison of methods, Cluster randomised trial, Small number of clusters

## Abstract

**Background:**

Cluster randomised trials (CRTs) are often designed with a small number of clusters, but it is not clear which analysis methods are optimal when the outcome is binary. This simulation study aimed to determine (i) whether cluster-level analysis (CL), generalised linear mixed models (GLMM), and generalised estimating equations with sandwich variance (GEE) approaches maintain acceptable type-one error including the impact of non-normality of cluster effects and low prevalence, and if so (ii) which methods have the greatest power. We simulated CRTs with 8–30 clusters, altering the cluster-size, outcome prevalence, intracluster correlation coefficient, and cluster effect distribution. We analysed each dataset with weighted and unweighted CL; GLMM with adaptive quadrature and restricted pseudolikelihood; GEE with Kauermann-and-Carroll and Fay-and-Graubard sandwich variance using independent and exchangeable working correlation matrices. P-values were from a t-distribution with degrees of freedom (DoF) as clusters minus cluster-level parameters; GLMM pseudolikelihood also used Satterthwaite and Kenward-Roger DoF.

**Results:**

Unweighted CL, GLMM pseudolikelihood, and Fay-and-Graubard GEE with independent or exchangeable working correlation matrix controlled type-one error in > 97% scenarios with clusters minus parameters DoF. Cluster-effect distribution and prevalence of outcome did not usually affect analysis method performance. GEE had the least power. With 20–30 clusters, GLMM had greater power than CL with varying cluster-size but similar power otherwise; with fewer clusters, GLMM had lower power with common cluster-size, similar power with medium variation, and greater power with large variation in cluster-size.

**Conclusion:**

We recommend that CRTs with ≤ 30 clusters and a binary outcome use an unweighted CL or restricted pseudolikelihood GLMM both with DoF clusters minus cluster-level parameters.

**Supplementary Information:**

The online version contains supplementary material available at 10.1186/s12874-022-01699-2.

## Background

Cluster randomised trials (CRTs) are often designed with a small number of clusters [1], but it is not clear which analysis methods are optimal when the outcome is binary.

In a CRT, groups of individuals known as clusters, such as health clinics or villages, are randomly assigned to receive either a control or intervention condition. Observations from the same clusters are likely to be more similar to one another than observations from different clusters, and it is well known that this correlation needs to be taken into account to prevent confidence intervals that are too narrow and p-values that are too small [2].

There are three broad types of analysis that can be used for CRTs: cluster-level analyses, generalised linear mixed effect models (GLMM), and generalised estimating equations with sandwich standard errors (GEE). In a cluster-level analysis, the observations from each cluster are summarised, and these cluster-level summaries are analysed using simple methods for independent data, most commonly with a weighted or unweighted t-test. In a GLMM, the correlation of observations in the same cluster is directly modelled by including a random effect for cluster. GEE assume a working correlation structure for observations in the same cluster, and standard errors are calculated allowing for the observed correlations in the data.

There is a wealth of literature on each type of analysis method with a small number of clusters. Cluster-level analysis is known to maintain control of type-one error with a small number of clusters and non-normally distributed outcomes [3, 4]. GLMM or GEE require small sample corrections with a small number of clusters to maintain an acceptable type-one error rate [5–8]. GLMM requires use of restricted maximum likelihood and comparison of test statistics to a t-distribution rather than the normal distribution [5]. GEE require use of a bias corrected standard error as well as use of a t-distribution. There have been many bias corrected standard errors developed and each performs well in different scenarios. Some are known to be conservative in scenarios common to CRTs [9], while others have closer to nominal type one error [7, 8, 10]. Continuous outcomes and binary outcomes with a high prevalence are well studied [6, 10, 11], and to date, all assessments have assumed model assumptions are met. Cluster-level analysis is known to maintain control of type-one error with non-normally distributed outcomes [3, 4]. GLMM are robust to some degree of non-normality [12], but this has not been explored for a small number of clusters or for GEE. With a large number of clusters, individual-level analysis with GLMM or GEE has greater power than a cluster-level analysis with varying cluster size [2]. Power is known to be reduced by use of the GEE small sample corrections with continuous outcomes [11], but this has not been studied for a binary outcome. We provide more detail of this previous literature for each type of analysis in the “Background to methods” section below.

Binary outcomes are the most common type of outcome for CRTs [1], but raise problems for the CRT analysis methods. Cluster-level methods become more challenging when some clusters have no events of interest, and GLMM require numerical methods of integration of the random effects [2]. Commonly used effect measures such as the odds ratio are also non-collapsible, so that GEE estimates have a different interpretation to cluster-level analysis and GLMM.

In this paper, we address some remaining gaps in the literature for binary outcomes: Is it possible to control type-one error for each method with a low prevalence outcome? If type-one error can be controlled, which type of method has greatest power? How sensitive is each type of analysis to non-normality of cluster effects? We begin by describing the analysis methods in more detail and reviewing previous literature on use of these methods with a small number of clusters for the estimation of an odds ratio. We then report an extensive simulation study that addresses our three research questions. We demonstrate the impact of analysis choice on an illustrative CRT and provide recommendations to trialists.

## Methods

### Background to analysis methods

In this section, we will review the analysis methods that have been shown to maintain nominal type-one error for CRTs with a binary outcome and a small number of clusters. We only consider analyses that do no adjust for covariates.

The estimates from these methods have different interpretations. The cluster-level analysis and GLMM provide cluster-specific intervention effect estimates; these are estimates of the average effect comparing one participant given the intervention to one participant given the control drawn from the same cluster. GEE provide population-average (also known as marginal) intervention effect estimates; these are estimates of the average effect comparing one participant given the intervention to one participant given the control drawn from the population.

We consider a CRT with $n$ clusters randomised to either an intervention or control condition with outcomes ${y}_{ijk}=\mathrm{0,1}$ in arm $i$ in cluster $j$ for individual $k=1,\dots ,{m}_{ij}$ where ${m}_{ij}$ is the number of observations in arm $i$ in cluster $j$.

#### Cluster-level analysis

In a cluster-level analysis, individual observations are summarised for each cluster. With a binary outcome, a proportion is commonly used, but for comparability with GLMM and GEE, here we will consider the log odds of the outcome in each cluster, so that we estimate an odds ratio intervention effect. In clusters with no events of interest, the log odds are not defined. To avoid this, we added 0.5 events and 0.5 non-events to each cluster [2], so that the cluster log odds are defined as:

$${s}_{ij}=\frac{{\sum }_{k=1}^{{m}_{ij}}{y}_{ijk}+0.5}{{m}_{ij}-{\sum }_{k=1}^{{m}_{ij}}{y}_{ijk}+0.5}$$

These cluster log-odds are independent of one another, so simple analysis procedures can then be used to derive a confidence interval and *p*-value. A t-test with degrees of freedom as n – 2 has been shown to maintain type-one error with as few as 6 clusters [13].

The t-test is known to be robust to relatively large deviations from the assumption of normality of cluster summaries [14]; supporting Fig. 1A and C show examples of non-normally distributed outcomes that do not affect t-test performance. However, the method becomes inefficient if clusters vary in size [15]. To improve the efficiency, several weights for the clusters and use of a weighted t-test have been suggested, but the performance of these weights remains unclear. While weighting clusters could maximise the use of information, this has to be considered against the uncertainty in the estimation of the weights themselves [2].

Here, we compare the performance of the following cluster weights:

##### Unweighted analysis

All clusters are given the same weight.

##### Inverse-variance weights

Inverse variance weights account for the information provided by each cluster by weighting each cluster inversely to the variance of the cluster summary. From a Taylor series expansion, the variance of the cluster log-odds is approximated by

$$Var\left(\mathrm{log}\left[\frac{{\widehat{p}}_{ij}}{1-{\widehat{p}}_{ij}}\right]\right)\approx {\sigma }_{b}^{2}+E\left[\frac{1}{{p}_{ij}\left(1-{p}_{ij}\right){m}_{ij}}\right]$$

where ${\widehat{p}}_{ij}$ is the observed proportion of observations with the outcome in arm $i$ in cluster $j$, ${\sigma }_{b}^{2}$ is the variance of the true cluster log-odds, ${p}_{ij}$ is the true proportion of observations with the outcome in arm $i$ in cluster $j$.

We can substitute the intracluster correlation coefficient (ICC) into this formula. The ICC is the between cluster variability divided by the total variability. We use the definition for the ICC on the log odds scale of ${\rho }_{i}={\sigma }_{b}^{2}/\left({\sigma }_{b}^{2}+1/{p}_{i}[1-{p}_{i}]\right)$ [16] where ${p}_{i}$ is the prevalence of the outcome in each arm.The variance of the cluster log-odds leads to different inverse-variance weights depending on assumptions about the prevalence of the outcome within clusters. Under a null hypothesis of the same mean prevalence in both arms, then the ICC is the same for both arms so that ${\rho }_{0}={\rho }_{1}=\rho$, and we get inverse-variance weights suggested by Kerry and Bland [15] (see supplementary text for derivation)

$${w}_{ij}=\frac{{m}_{ij}}{1+\left({m}_{ij}-1\right)\rho }$$

Identical weights can also be derived using a different definition for the ICC that assumes that there is an underlying latent variable that determines whether each individual experiences the outcome. This latent variable is assumed to follow the logistic distribution [16].

Weighting by cluster size and within-cluster variance have also been used elsewhere but are not considered here. Weighting by cluster size ignores the non-linear impact of cluster size on the information provided by a cluster and has been shown to give biased effect estimates [11, 17]. Weighting by within-cluster variance ignores the between cluster element of the cluster log-odds variance and has been shown to give inflated type-one error unless the outcome is very common [17].

#### Generalised linear mixed effect models

GLMMs with a binomial distribution and logit link directly model the between cluster variation so that:

$$E[{\varvec{y}}|{\varvec{X}},{\varvec{u}}]=\frac{exp\left({\varvec{X}}{\varvec{\beta}}+{\varvec{u}}\right)}{1+exp\left({\varvec{X}}{\varvec{\beta}}+{\varvec{u}}\right)}$$

where ${\varvec{y}}$ is a vector of outcomes of length $n{m}_{ij}$, ${\varvec{X}}$ is a $n{m}_{ij}$ x $2$ matrix of fixed effect covariates consisting of a vector of ones and the trial arm assignment, ${\varvec{\beta}}$ is a vector of fixed-effect parameters consisting of the intercept (${\beta }_{0}$) and log odds ratio comparing control and intervention conditions (${\beta }_{1}$), and ${\varvec{u}}$ is a vector of random effects for clusters with elements ${u}_{ij}\sim N(0,{\sigma }_{b}^{2})$.

Maximum likelihood is used to estimate the parameters ${\varvec{\beta}}$, and ${\sigma }_{b}^{2}$. For CRTs with a continuous outcome, a similar GLMM is used, but with a normal distribution with identity link. Maximum likelihood estimation leads to biased estimation of ${\sigma }_{b}^{2}$ with a small number of clusters, and use of restricted maximum likelihood estimation, which applies a transformation to the data to remove fixed effects before estimating random effect variances, reduces this bias [18].

For the binary outcome model above, the marginal likelihood does not have a closed form expression, so numerical integration methods are required. Adaptive quadrature is a commonly used technique, but there is no equivalent restriction technique to the one used to reduce bias with continuous outcomes. Pseudo-likelihood [19] and penalised quasi-likelihood [20] perform less well than adaptive quadrature with a large number of clusters [20], but methods of restriction are available. Elff et al. found that this made penalised quasi-likelihood a more suitable technique for data with fewer clusters with a common outcome and probit link [5].

In addition to selection of integration method, confidence intervals and p-values should be constructed with a t-distribution when the number of clusters is small to account for uncertainty in estimation of the standard error. There are three commonly used options for the degrees of freedom:

- Clusters minus cluster-level parameters. In an unadjusted analysis, this is $D{F}_{C-P}=n-2$
- Satterthwaite [21]. For a test of the intervention effect parameter, degrees of freedom are
  $$D{F}_{S}=\frac{2Var{({\beta }_{1})}^{2}}{Var\left[Var({\beta }_{1})\right]}$$

Where $Var\left[Var({\beta }_{1})\right]$ is approximated using the multivariate delta method.

- Kenward and Roger [22] developed a small sample correction that involves inflation of the standard error as well as a degree of freedom correction (DF_KR_). For a test of the intervention effect parameter, the degrees of freedom part of this correction are the same as DF_S_, but the standard error may be different.

With continuous outcomes, Satterthwaite degrees of freedom give closest to nominal type-one error, and clusters minus cluster-level parameters and Kenward-Rogers degrees of freedom are more conservative [11]. With binary outcomes, clusters minus cluster-level parameters has previously given closest to nominal coverage [6] with a common outcome.

With a large number of clusters, GLMM provide similar results with normally and non-normally distributed cluster effects (here these are cluster log-odds) except for extreme cases of non-normality or very large between cluster variability [12, 23]. Supporting Fig. 1B and C shows examples of cluster effect distributions that have not affect GLMM performance. However, this has not been studied for settings with a small number of clusters.

#### Generalised Estimating Equations

GEE model the marginal individual level data treating the correlation parameters as nuisance parameters. We assume a correlation structure for the data, which gives a covariance matrix ${{\varvec{V}}}_{{\varvec{W}}{\varvec{i}}{\varvec{j}}}$ for the vector of outcomes in each cluster ${{\varvec{y}}}_{{\varvec{i}}{\varvec{j}}}$. We use the logit link so that

$${\varvec{\mu}}=E[{\varvec{y}}|{\varvec{X}}]=\frac{exp\left({\varvec{X}}{\varvec{\beta}}\right)}{1+exp\left({\varvec{X}}{\varvec{\beta}}\right)}$$

The (uncorrected) sandwich covariance matrix of $\widehat{{\varvec{\beta}}}$ is

$${{\varvec{V}}}_{{\varvec{s}}}={{\varvec{V}}}_{{\varvec{M}}}\left[{\sum }_{j=0}^{1}{\sum }_{i=1}^{n/2}{{\varvec{D}}}_{{\varvec{i}}{\varvec{j}}}{{\varvec{V}}}_{{\varvec{W}}{\varvec{i}}{\varvec{j}}}^{-1}\widehat{{\varvec{C}}{\varvec{o}}{\varvec{v}}}\left({{\varvec{y}}}_{{\varvec{i}}{\varvec{j}}}\right){{\varvec{V}}}_{{\varvec{W}}{\varvec{i}}{\varvec{j}}}^{-1}{{\varvec{D}}}_{{\varvec{i}}{\varvec{j}}}\right]{{\varvec{V}}}_{{\varvec{M}}}$$

where ${{\varvec{V}}}_{{\varvec{M}}}$ is the model based variance of ${\varvec{\beta}}$, ${{\varvec{D}}}_{ij}={\partial{\varvec{\mu}}}_{{\varvec{i}}{\varvec{j}}}/\partial{\varvec{\beta}}\boldsymbol{^{\prime}}$, and $\widehat{Cov}\left({{\varvec{y}}}_{{\varvec{i}}{\varvec{j}}}\right)=\left({{\varvec{y}}}_{{\varvec{i}}{\varvec{j}}}-{\widehat{{\varvec{\mu}}}}_{{\varvec{i}}{\varvec{j}}}\right){\left({{\varvec{y}}}_{{\varvec{i}}{\varvec{j}}}-{\widehat{{\varvec{\mu}}}}_{{\varvec{i}}{\varvec{j}}}\right)}^{T}$**.**

With fewer than 40–50 clusters [24], the sandwich covariance estimator used in conjunction with GEE are known to estimate standard errors that are too small on average, hence they are negatively biased, and several bias corrections have been suggested to reduce this bias [7–9, 25, 26]. Corrections developed by Kauermann and Carroll [8] and Fay and Graubard [7] have had particularly promising performance across a range of scenarios where CRTs are used [10, 27]. Others were often conservative [9] or highly variable [26].

Kauermann and Carroll suggested the estimator: [8]

$${{\varvec{V}}}_{{\varvec{K}}{\varvec{C}}}={{\varvec{V}}}_{{\varvec{M}}}\left[{\sum }_{j=0}^{1}{\sum }_{i=1}^{n/2}{{\varvec{D}}}_{{\varvec{i}}{\varvec{j}}}{{\varvec{V}}}_{{\varvec{W}}{\varvec{i}}{\varvec{j}}}^{-1}{{\varvec{A}}}_{{\varvec{K}}{\varvec{C}}{\varvec{i}}{\varvec{j}}}\widehat{{\varvec{C}}{\varvec{o}}{\varvec{v}}}\left({{\varvec{y}}}_{{\varvec{i}}{\varvec{j}}}\right){{\varvec{A}}}_{{\varvec{K}}{\varvec{C}}{\varvec{i}}{\varvec{j}}}{{\varvec{V}}}_{{\varvec{W}}{\varvec{i}}{\varvec{j}}}^{-1}{{\varvec{D}}}_{{\varvec{i}}{\varvec{j}}}\right]{{\varvec{V}}}_{{\varvec{M}}}$$

where $${{\varvec{A}}}_{{\varvec{K}}{\varvec{C}}{\varvec{i}}{\varvec{j}}}={\left[{{\varvec{I}}}_{{\varvec{i}}{\varvec{j}}}-{{\varvec{D}}}_{{\varvec{i}}{\varvec{j}}}{{\varvec{V}}}_{{\varvec{M}}}{{\varvec{D}}}_{{\varvec{i}}{\varvec{j}}}^{{\varvec{T}}}{{\varvec{V}}}_{{\varvec{W}}{\varvec{i}}{\varvec{j}}}^{-1}\right]}^{-1/2}$$.

Fay and Graubard suggested the estimator: [7]

$${{\varvec{V}}}_{{\varvec{F}}{\varvec{G}}}={{\varvec{V}}}_{{\varvec{M}}}\left[{\sum }_{j=0}^{1}{\sum }_{i=1}^{n/2}{{\varvec{A}}}_{{\varvec{F}}{\varvec{G}}{\varvec{i}}{\varvec{j}}}{{\varvec{D}}}_{{\varvec{i}}{\varvec{j}}}{{\varvec{V}}}_{{\varvec{W}}{\varvec{i}}{\varvec{j}}}^{-1}\widehat{{\varvec{C}}{\varvec{o}}{\varvec{v}}}\left({{\varvec{y}}}_{{\varvec{i}}{\varvec{j}}}\right){{\varvec{V}}}_{{\varvec{W}}{\varvec{i}}{\varvec{j}}}^{-1}{{\varvec{D}}}_{{\varvec{i}}{\varvec{j}}}{{\varvec{A}}}_{{\varvec{F}}{\varvec{G}}{\varvec{i}}{\varvec{j}}}\right]{{\varvec{V}}}_{{\varvec{M}}}$$

where $${{\varvec{A}}}_{{\varvec{F}}{\varvec{G}}{\varvec{i}}{\varvec{j}}}=diag\left[\left(1-min\left[0.75,{{\varvec{D}}}_{{\varvec{i}}{\varvec{j}}}{{\varvec{V}}}_{{\varvec{W}}{\varvec{i}}{\varvec{j}}}^{-1}{{\varvec{D}}}_{{\varvec{i}}{\varvec{j}}}^{{\varvec{T}}}{{\varvec{V}}}_{{\varvec{M}}}\right]\right)\right]$$

As well as standard error corrections, it is necessary to construct confidence intervals and p-values from a t-distribution as has been seen for cluster-level analysis and GLMM. There is less literature for GEE, but DF_C-P_ has performed well [10].

Unlike the cluster level analysis and GLMM, GEE do not make any assumptions about the distribution of the cluster log-odds. They were developed as a robust method of analysis for non-normal outcomes, so we expect this method to be robust to non-normality of cluster log-odds.

### Comparison between these methods

There is particularly sparse literature comparing the different types of methods. Since it may now be possible to maintain type-one error with a small number of clusters for all approaches, comparisons of power are relevant. While with a large number of clusters, it is known that the individual-level methods we’ve described are more powerful than the cluster-level method when clusters vary in size [28], there are few comparisons of power with small sample corrections applied to the individual-level methods. For continuous outcomes and normally distributed cluster-level summaries, GLMMs with a small sample correction had a higher power than unweighted cluster-level analyses and GEEs, and a similar power as inverse variance weighted cluster-level analyses [11]. Others have found that GLMM had higher power than GEE after small sample corrections had been applied [29].

### Simulation study methods

We conducted a simulation study to compare the performance of the analysis methods described above. The simulations were performed in SAS software, version 9.4 of the SAS system for windows[30]. The scenarios included in the simulations are given in table 1 and more details are in the supporting information text. All combinations of each scenario were simulated with 1000 repetitions so that there is a 95% probability that true type-one error of 5% is estimated to be between 3.6% and 6.4%.

**Table 1:** Summary of simulation study scenarios

Parameter	Number of scenarios	Values
**Total number of clusters**	4	8, 12, 20, 30
**Mean Cluster size (**$\overline{{\varvec{m}} }$**)**	3	10, 50, 1000
**Coefficient of variation (CV) of cluster size**	3	$$CV=\frac{s}{\overline{m} }= 0, 0.5, 0.8$$ Where s is the standard deviation in cluster sizes and$$\overline{m }$$is the mean cluster size, Cluster size $${m}_{ij}$$is sampled from a negative binomial distribution as follows: $$\delta \sim Negbin\left(no of fails=\frac{{(\overline{m }-2)}^{2}}{{s}^{2}-(\overline{m }-2)},p of fail=\frac{\overline{m }-2}{{s}^{2}}\right)$$ $${m}_{ij}=2+\delta$$
**Control cluster prevalence**	2	10%, 30%
**Intervention effect**	2	No effect, or odds ratio between 1.12 and 11.49 selected for each scenario to achieve 80% power
**ICC**	4	0.001, 0.01, 0.05, 0.1
**Cluster effect distribution**	3	Normal: ${u}_{ij}\sim N(0,{\sigma }_{b}^{2})$ Gamma ${u}_{ij}=\frac{{\sigma }_{b}\left({a}_{ij}-2\right)}{\sqrt{2}}$where ${a}_{ij}\sim Gamma\left(\mathrm{2,1}\right)$ Uniform ${u}_{ij}\sim Uniform\left(-\sqrt{3{\sigma }_{b}^{2}},\sqrt{3{\sigma }_{b}^{2}}\right)$ Distributions are defined to give the specified between cluster variability set by the ICC

#### Data generating mechanism

We simulated binary, clustered data using the data generating model:

$${Y}_{ij}\sim Binomial\left(\frac{exp\left[{\beta }_{0}+{\beta }_{1}i+{u}_{ij}\right]}{1+exp\left[{\beta }_{0}+{\beta }_{1}i+{u}_{ij}\right]}, {m}_{ij}\right)$$

where ${Y}_{ij}$ is the number of events in arm $i$ in cluster $j$, ${\beta }_{0}$ is the true log-odds of the outcome in the control condition, ${\beta }_{1}$ is the log odds ratio intervention effect, and ${u}_{ij}$ is a random effect for cluster with mean zero and variance ${\sigma }_{b}^{2}$.

The prevalence of the outcome in the control condition was either 10% or 30%. We simulated scenarios with and without an intervention effect (${\beta }_{1}$). For the scenarios with an intervention effect, we used the Stata 15 [31] power command to select cluster-specific odds ratios that would be expected to have 80% power for each scenario. This command uses the design effect $1+\left(m-1\right)\rho$ to account for clustering and the design effect of van Breukelen, Candel, and Berger to account for unequal cluster size [32].

The ICC was set to 0.001, 0.01, 0.5, or 0.1 on the log odds scale, defined as ${\sigma }_{b}^{2}/({\sigma }_{b}^{2}+{\pi }^{2}/3)$, to span a range of common values in health research [33–35]. For the distribution ${u}_{ij}$, we considered a normal distribution, a uniform distribution to explore the impact of kurtosis, and a gamma distribution with shape parameter $\lambda =2$. These distributions were selected as they are the limit for which GLMMs estimate unbiased cluster-level coefficients with a large number of clusters [12, 23].

#### Trial designs

We simulated trials with a total of 8, 12, 20, or 30 clusters and a 1:1 randomisation ratio. Cluster size was either common to all clusters or simulated to vary between clusters. Variable cluster sizes were drawn from a negative binomial distribution to give a minimum cluster size of 3 and coefficient of variation in cluster size of 0.5 (the median CV of UK primary care trust size [36]), or 0.8 (a large variability) [11, 37]. The mean cluster size was either 10, 50, or 1000 to represent small, medium and very large clusters [1].

#### Estimand and Analysis Methods

The estimands of interest for the analysis of the simulated trials was the odds ratio intervention effect and the statistical test for no intervention effect. We use a cluster specific estimand for the cluster-level analysis and GLMM and a population-averaged estimand for the GEE.

We analysed the data with all methods previously described: unweighted cluster analysis (CL-UNW), and inverse variance weights with equal variance within clusters (CL-W); GLMM using adaptive Gauss-Hermite quadrature (AQ) or restricted pseudo-likelihood (REPL) and degrees of freedom as clusters minus cluster-level parameters (DF_CP_), Satterthwaite (DF_S_), or Kenward-Rogers (DF_KR_) where DF_S_ and DF_KR_ were only available for REPL; and GEE with a sandwich variance with the Kauermann and Carroll correction (KC) or Fay and Graubard correction (FG) with boundary parameter 0.75 using DF_CP_ and an independent (I) or exchangeable (E) working correlation matrix. The exchangeable working correlation matrix GEE were only run on scenarios with mean cluster size of 10 or 50 due to unfeasibly long run time with a cluster size of 1000.

Our data generating mechanism specified a cluster-specific intervention effect odds ratio. Since GEE estimate population-averaged (marginal) odds ratios, we estimated the true marginal effects to compare to GEE estimates using the approximate formula [38]:

$${\beta }_{Marginal}\approx {\beta }_{Conditional}{\left({\left[\frac{16\sqrt{3}}{15\pi }\right]}^{2}{\sigma }_{b}^{2}+1\right)}^{-1/2}$$

First we select the cluster-level, GLMM, and GEE method that has most consistently controls type-one error in the simulation study; then we compare the performance of these three methods.

#### Performance measures

For each scenario and analysis method, we calculated the standardised bias of the intervention effect estimate, which is the bias as a percentage of the standard deviation of the intervention effect estimates across the 1000 repetition; relative bias in standard errors; type-one error; and power [39]. We also calculated coverage but results are not shown due to the similarity of results to type-one error results. We refer to type-one error less than 3.6% as conservative and type-one error above 6.4% as inflated. Convergence rates were summarised and analyses that did not converge were excluded.

## Results

For each type of analysis, we will summarise important results, more detail is given in the supporting information.

### Intervention effect estimate bias, standard error bias, and type-one error

#### Cluster-level methods

Less than 1% of CL-UNW or CL-W failed to provide intervention effects or p-values in all scenarios. Non-convergence only occurred if all clusters-level summaries were identical within each arm, giving a within-arm variance of zero.

**Fig. 1:**
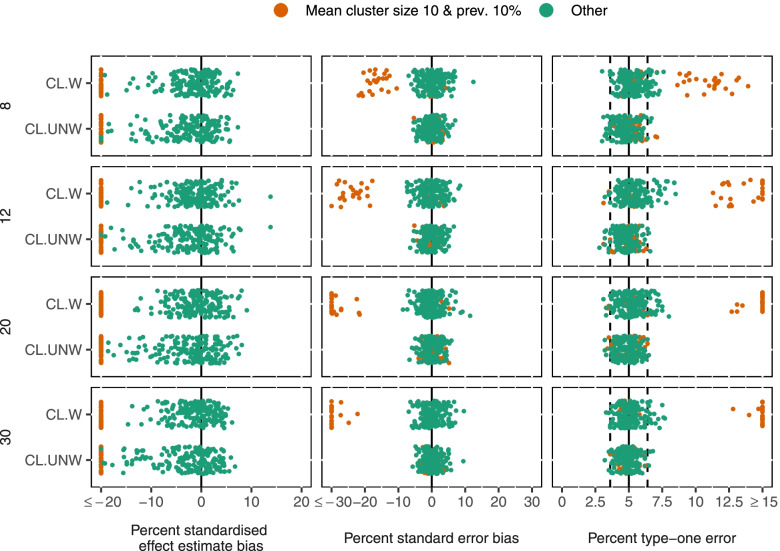
Performance measures of cluster-level analysis methods by number of clusters (rows), cluster size and outcome prevalence (colour). Measures shown (columns): Standardised intervention effect estimate bias, standard error bias, type-one error. Each dot represents a scenario summarised over the 1000 repetitions. All 864 scenarios are shown for each measure

CL-UNW and CL-W had mean intervention effect estimate standardised bias of 52% and 38% closer to the null respectively across scenarios with mean cluster size 10 and low outcome prevalence (Fig. 1). Both methods demonstrated no bias in any other scenario (mean standardised bias -2% for CL-UNW and -1% for CL-W).

Standard errors of CL-UNW were within 10% of the standard deviation of simulated estimates in all scenarios, and type-one error was close to nominal in all scenarios (Fig. 1). Confidence interval coverage was often low with mean cluster size 10 and low outcome prevalence (102/144(71%) scenarios had coverage < 93.6%) due to the biased intervention effect estimate.

CL-W standard errors were between 46% smaller and 6% larger than the standard deviation of estimates with mean cluster size 10 and low prevalence. Standard errors were closer to the standard deviation of simulated estimates in other scenarios (between 9% smaller and 14% larger). Type-one error for CL-W was inflated with mean cluster size 10 and low outcome prevalence (97/144(67%) scenarios with type-one error > 6.4%) and when cluster size varied (type-one error > 6.4% in 25/240(10%) scenarios with cluster size CV = 0.5, and 49/240(20%) with cluster size CV = 0.8).

Supporting Figs. 2, 3, 4, 5 and 6 show cluster-level analysis performance by each simulation study parameter.

#### GLMM

In both REPL and AQ, up to 10% of models failed to converge with 8 clusters with mean cluster size of 10 and low outcome prevalence. REPL resulted in up to 8% non-convergence with mean cluster size 1000 and ICC = 0.001; this was more pronounced with 30 clusters but persisted with 20 clusters. Non-convergence was less than 5% in all other scenarios.

**Fig. 2:**
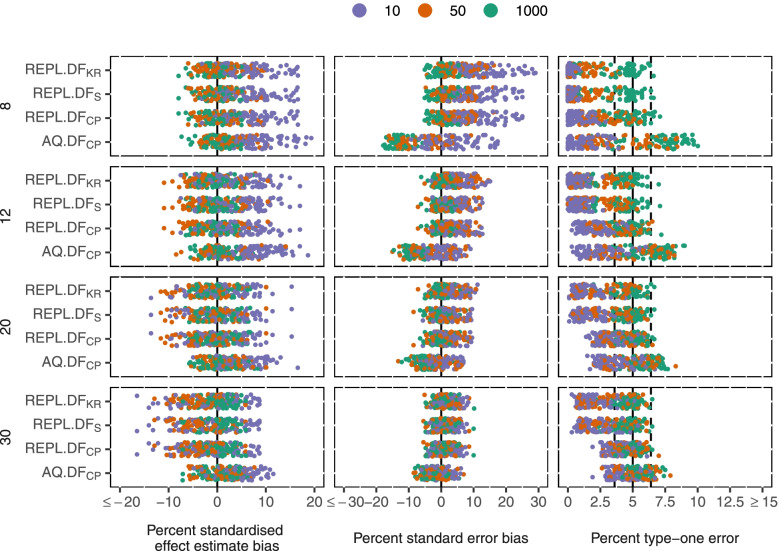
Performance measures of GLMM methods by number of clusters (rows), and mean cluster size (colour). Measures shown (columns): Standardised intervention effect estimate bias, standard error bias, type-one error

Both REPL and AQ gave estimates of the intervention effect with minimal bias in all scenarios (mean 2.9% standardised bias for AQ and 0.6% for REPL across all scenarios, Fig. 2).

AQ resulted in standard errors that were too small with mean cluster size 1000 with 20 or fewer clusters (mean 8% smaller than the standard deviation of simulations with 12 clusters and 12% with 8 clusters). Bias in standard errors increased with larger ICC (ICC = 0.1 standard error bias = -6%, ICC = 0.001, standard error bias = -3%). This led to mean type-one error of 7% with ICC = 0.1, mean cluster size of 1000 and 8 or 12 clusters.

REPL gave more consistent standard errors. However, with mean cluster size 10, standard errors were a mean 4% larger than the standard deviation of simulations with 12 cluster and 10% larger with 8 clusters. The Kenward-Roger standard error correction had minimal impact. Combining REPL with DF_CP_ controlled type-one error, but was conservative in the scenarios where the standard errors were inflated (mean cluster size 10 had mean type-one error = 3.8% with 30 clusters, 1.9% with 12 clusters, and 0.8% with 8 clusters). DF_KR_ and DF_SA_ were more conservative.

Supporting Figs. 7, 8, 9 and 10 show GLMM methods performance by each simulation study parameter.

#### GEE

With an independent working correlation matrix, in most scenarios, less than 3% failed to converge. Up to 9% failed to converge with mean cluster size 10 and outcome prevalence 10%. With an exchangeable working correlation matrix, non-convergence was common with varying cluster size. With cluster size CV = 0.8 and mean cluster size 10, a mean of 20% failed to converge; with mean cluster size 50, this increased to a mean of 34% failing to converge.

**Fig. 3:**
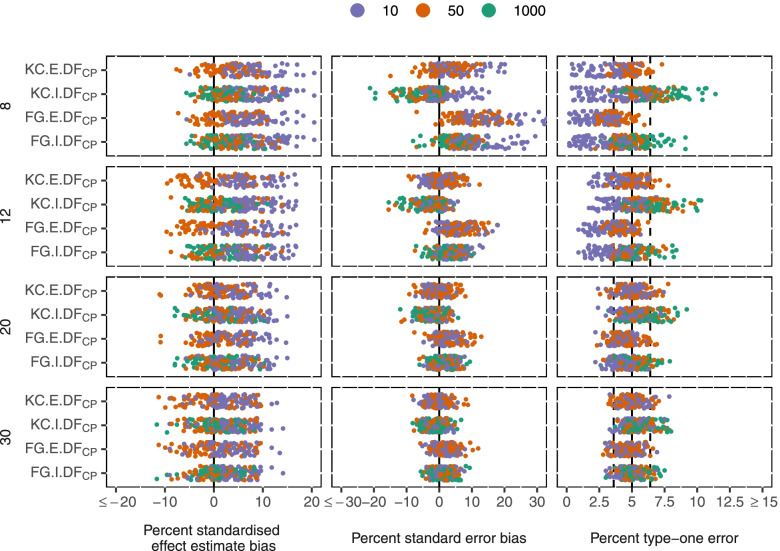
Performance measures of GEE methods by number of clusters (rows), and mean cluster size (colour). Measures shown (columns): Standardised intervention effect estimate bias, standard error bias, type-one error

The intervention effect was estimated with little bias where no effect was present and negligible bias (compared to estimated marginal effects) when the intervention did have an effect in truth (mean 5% and 3% standardised bias for independent and exchangeable working correlation matrices respectively, Fig. 3). Variability of effect estimates was similar between the independent and exchangeable working correlation matrices.

With an independent working correlation matrix, both KC and FG standard errors demonstrated little bias with 20 or more clusters. With 12 or fewer clusters, FG standard errors were on average 6% too large and KC standard errors were on average 2% too small (Fig. 3). With FG standard errors, type-one error was conservative in 25% of scenarios and inflated in 3% of scenarios. Inflated type-one error occurred when clusters were large and there was large variability in cluster size.

With an exchangeable working correlation matrix, FG standard errors became more variable and had a mean 10% overestimation of standard errors with 12 or fewer clusters. KC standard errors had little bias with 12 or more clusters and were a mean 3% overestimated with 8 clusters. With FG standard errors, type-one error was conservative in 33% of scenarios and inflated in 1% of scenarios.

In order to select a working correlation matrix to take forward to compare with GLMM and CL methods, we also looked at power. With FG standard errors and DF_CP_, power was similar with an independent or exchangeable working correlation matrix (mean 1% greater power with an independent working correlation matrix). Power was similar with 20–30 clusters (exchangeable mean 0.4% higher) but favoured an independent working correlation matrix with fewer cluster (exchangeable mean 0.8% and 3.8% lower with 12 and 8 cluster respectively). Power favoured an independent working correlation matrix with ICC = 0.001 (mean 3.1% higher power than exchangeable) but favoured an exchangeable working correlation matrix with ICC = 0.1 (mean 1.6% higher power with exchangeable).

Due to the similar power and better convergence, we carried an independent working correlation matrix forward to the comparison with the cluster-level method and GLMM.

Supporting Figs. 11, 12, 13 and 14 show GEE method performance by each simulation study parameter.

#### Comparison of cluster-level method, GLMM, and GEE

Next we compare the best performing method from each analysis type: CL-UNW, REPL.DF_CP_, and GEE with FG standard errors and an independent working correlation matrix (FG.I.DF_CP_).

**Fig. 4:**
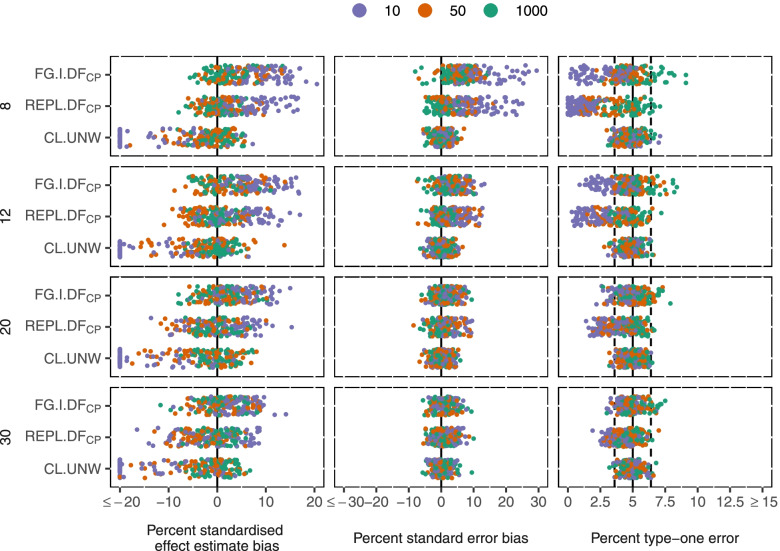
Comparison of bias and type-one error of unweighted cluster-level analysis, GLMM with REPL and DF_CP_, and GEE with FG standard errors and DF_CP_ by number of clusters (rows), and mean cluster size (colour). Measures shown (columns): Standardised intervention effect estimate bias, standard error bias, type-one error

All three method controlled type-one error in the majority of scenarios (Fig. 4). CL-UNW controlled type-one error most consistently; only 1% of scenarios had inflated and 5% conservative type-one error. REPL-BW has the most conservative type-one error: 42% of scenarios had conservative type-one error and 1% has inflated type-one error. FG.I.DF_CP_ had the most variable type-one error: 6% of scenarios had inflated type-one error and 17% conservative.

### Power

**Fig. 5:**
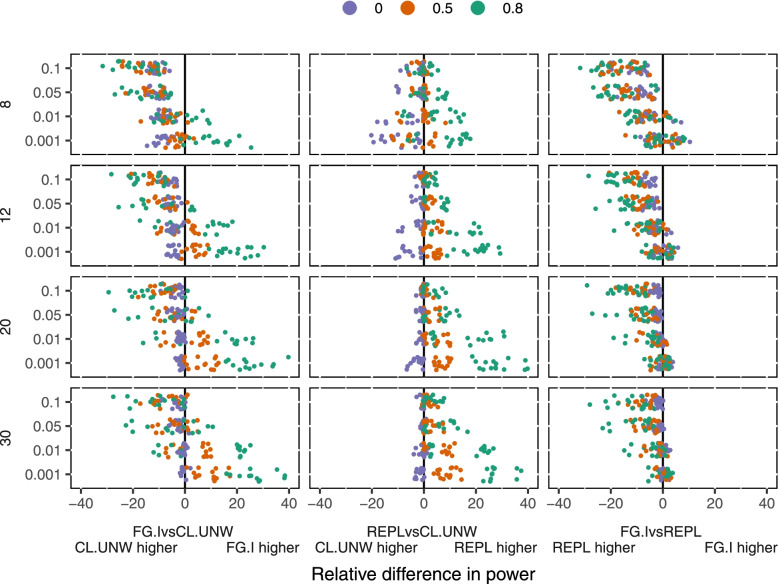
Power comparison of unweighted cluster-level analysis (CL.UNW), GLMM with REPL and DF_CP_ (REPL), and GEE with FG standard errors and DF_CP_ (FG.I) (columns) by number of clusters (rows), ICC (y axis), and variability of cluster size (colour)

Excluding scenarios with low prevalence and cluster size of 10 (due to biased effect estimates from CL-UNW), REPL.DF_CP_ has on average 2% higher power than CL-UNW, and FG.I.DF_CP_ had on average 3% lower power than CL-UNW (Fig. 5).

The power difference between CL-UNW and FG.I.DF_CP_ was most strongly influenced by the ICC with higher ICC leading to CL-UNW having higher power: with ICC = 0.1 CL-UNW had a mean 10% higher power than FG.I.DF_CP_, but with ICC = 0.001, FG.I.DF_CP_ had a mean 8% greater power than CL-UNW. With fewer clusters, power favoured CL-UNW.

The power difference between REPL.DF_CP_ and CL-UNW was most strongly influenced by variation in cluster size. With common cluster size, CL-UNW had a mean 2% higher power than REPL.DF_CP_; with CV = 0.5, REPL-BW had a mean 2% higher power than CL-UNW; and with CV = 0.8, REPL.DF_CP_ had a mean 10% higher power than CL-UNW. With fewer clusters, power became more similar between the methods. With higher ICC, power become more similar between the methods.

Across all scenarios (including scenarios with low prevalence and cluster size of 10), REPL.DF_CP_ had a mean 5% higher power than FG.I.DF_CP_. This was most strongly influenced by ICC: with ICC = 0.1, REPL.DF_CP_ had a mean 10% higher power, but with ICC = 0.001, REPL.DF_CP_ and FG.I.DF_CP_ had similar power. Number of clusters had minimal impact on the difference in power.

Supporting Figs. 15, 16 and 17 show power comparisons by each simulation study parameter.

### Robustness to non-normality

There was no difference in our findings based on whether the clusters log-odds were distributed normally, or with skew or kurtosis (supporting Figs. 3, 8, 12, and 16).

### Recommendations

Table 2 summarises our results to provide recommendations of the most robust and powerful analysis by scenario.

**Table 2:** Summary of simulation study results and recommendations on their use

	**Cluster-Level Method**	**GLMM**	**GEE**
**Method**	Use equal weighting of clusters and clusters minus cluster-level parameters degrees of freedom	Use restricted pseudo-likelihood and clusters minus cluster-level parameters degrees of freedom	Use Fay and Graubard standard errors, clusters minus cluster-level parameters degrees of freedom, and an independent working correlation matrix
**Valid results **^a^	Cluster size > 10 Or Common outcome (prevalence > 10%)	All scenarios	Cluster size ≤ 50 Or CV cluster size ≤ 0.5
**Competitive power **^b^	Common cluster size Or High ICC (ICC > 0.05)	Varying cluster size Or 20 + clusters	Low ICC ≤ 0.01

^a^ Unbiased effects with controlled or conservative type-one error

^b^ The method/s with greatest or similar to greatest power in a scenario

## Illustrative example

Treatment for tuberculosis involves 6 months of alternate daily drugs. Recovery is hampered by non-adherence to treatment and the standard of care (SoC) is directly observed therapy where a health worker or family member directly observes the patient taking their medication. This is costly and has limited impact on adherence [40].

In one trial, two interventions, a text message reminder and electronic monitoring box for medication, were compared to SoC [41]. Here, we focus on the comparison of the monitoring box to SoC: a comparison with 9 clusters in each arm. Randomisation was stratified by province and whether clusters were urban: this is ignored for simplicity in this example. In the monitor box arm, patient’s medication was stored in a box that recorded openings of the box that clinicians could review to assess the need for adherence counselling, and a light and sound reminded patients to take medication. We will focus on a secondary outcome from the trial: whether patients missed more than 10% of doses over treatment. There was a mean 116 patients per cluster with coefficient of variation 0.1. The ICC on the log-odds scale was estimated as 0.09 (estimated from the REPL GLMM).

**Fig. 6:**
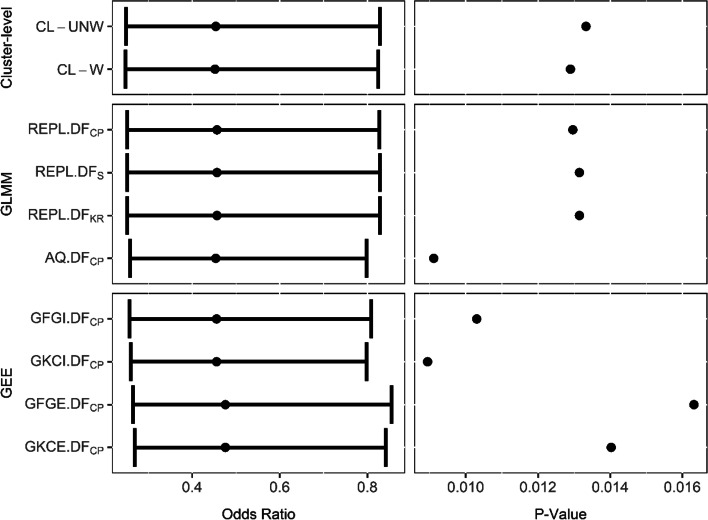
Motivating example results analysed by all methods considered in the simulation study. Left panel shows odds ratios and confidence intervals, right panel shows *p* values. Rows are analysis methods

Figure 6 shows the estimated intervention effects, confidence intervals, and p-values from each of the methods considered in this paper. The outcome was common in both arms: 59% and 41% in the control and intervention arms missed more than 10% of doses respectively. All analyses find strong evidence that the monitor box improved adherence compared to SoC, but the strength of evidence varied in line with the simulation study results.

Since clusters are medium sized, Table 2 recommends use of CL-UNW or REPL.DF_CP._ These provide almost identical results (CL-UNW: OR = 0.45 95% CI [0.25, 0.83] *p* = 0.013, REPL.DF_CP_: OR = 0.46 95% CI [0.25, 0.83] *p* = 0.013).

Since this example has small variability in cluster size, CL-W would be likely to have nominal type-one error, hence the similar result to CL-UNW. The inflated type-one error of AQ.DF_CP_ has resulted in a smaller *p*-value (*p* = 0.009) and narrower confidence interval (0.26, 0.80), but the degrees of freedom has had little impact on REPL as the clusters are not small in this trial. GEE methods may have inflated type-one error due to the cluster size, but were also found to have lower power than other methods. This has led to more variable results (*p* = 0.009 to 0.016).

## Discussion

We have identified methods to control the type-one error with as few as 8 clusters with cluster-level analysis, GLMM, and GEE in high and low prevalence settings. Cluster-level analysis should give equal weight to all clusters. GLMM should use REPL to integrate the likelihood, and GEE should use the small sample standard error correction from Fay and Graubard. All methods require a t-distribution with clusters minus cluster-level parameters as the degrees of freedom to calculate confidence intervals and *p*-values. We found that unweighted cluster-level analysis had greatest power with common cluster size and competitive power when the ICC was high. GLMMs using REPL had greatest power with varying cluster size or 20 or more clusters despite conservative type-one error. GEE with FG standard errors tended to have equal or lower power than the other methods. All methods performed well with non-normally distributed cluster effects.

Our comparison of cluster-level analysis methods identified problems with inverse-variance weighting of clusters. The weighted least squares method assumes that the weights are known, when in truth they are estimates. This leads to the bias in standard errors we observed with CL-W [42]. Use of a robust standard error may be able to account for weight estimation, but this is likely to lead to similar results to the GEE methods shown in this paper, which had lower power. Cluster-level analysis is simple to code manually in any software, and a user written Stata command clan is also available [43].

We found that the integration method used in the GLMM was important for obtaining unbiased standard errors: REPL outperformed AQ. This extends the findings of Elff et al [5] to logistic models and to low prevalence outcomes. We used the SAS glimmix procedure to implement this method. The R function glmmPQL implements a similar method [44]. To our knowledge, REPL is not available in the statistical software Stata. Our findings of nominal type-one error with clusters minus cluster-level parameters and conservative type-one error with Kenward-Rogers and Satterthwaite are supported by previous research [6], and we determine that these findings hold for a low prevalence outcome.

For GEE, our recommendation of FG standard errors with clusters minus cluster-level parameter degrees of freedom are in agreement with others [10, 45–47]. This previous literature has not reported rates of convergence, which we found were low in some scenarios with an exchangeable working correlation matrix. The poor convergence was likely due to computational complexity from inversion of a matrix the same size as the cluster size and some iterations requiring inversion of a not invertible matrix [48]. Our comparison of power by choice of working correlation matrix is novel. With a large number of clusters, correct choice of working correlation matrix is known to improve power [49]. We found that this difference diminished with a small number of clusters so that there was little benefit from fitting the more complex exchangeable working correlation matrix. Similar results have been seen for stepped wedge cluster randomised trials [50] with a cluster size of 50 or less, so our finding of similarity is likely do to the smaller cluster sizes used for this comparison. This method is widely implemented in statistical software: we used the glimmix procedure in SAS [30], in Stata a user written command xtgeebcv is available [51], and in R the saws package implements the FG correction [7].

Our comparison of power from the three types of analysis is novel. We found that despite conservative type-one error, GLMM generally had greater or competitive power compared to GEE or cluster-level analysis. Contrary to settings with a large number of clusters, we found that cluster-level analysis maintained competitive power when the ICC was large, even with varying cluster size. GEE had competitive power with a low ICC, but often lower power than GLMM. Low power was also identified by Leyrat et al. for continuous outcomes [11]. Where power and type-one error are similar between the methods, and convergence of GEE is reasonable, the choice could be guided by whether researchers are interested in estimating a cluster-specific or population-averaged intervention effect. Another consideration of this choice is non-normality of the cluster effects. We found no difference in the performance of methods with the distributions we considered. These were distributions where the limits of where mixed effect models perform well with a large number of clusters [12], so if a larger degree of non-normality is suspected, we recommend use of GEE or cluster-level analysis.

Our findings were similar regardless of the distribution of cluster log-odds for all numbers of clusters that we considered. Since our choice of non-normality was the boundary of good GLMM performance with a large number of clusters, this suggests a similar performance of methods with a small number of clusters to their performance with a large number of clusters [12, 23]. Therefore, a cluster-level analysis should be used with very skewed data, but either method remains suitable with some skew or data that shows kurtosis.

All our selected methods struggled with a mean 10 observations per cluster and low outcome prevalence, but this scenario of few clusters, that are small, and with low prevalence is unlikely to occur in practice. Unweighted cluster-level analysis gave biased intervention effect estimates in these scenarios due to the presence of clusters with no events. GLMM and GEE methods overestimated standard errors in these scenarios. With larger clusters, low prevalence generally had little impact on results.

Our simulation study covered a broad range of scenarios where CRTs are common. However, there are analysis methods that we did not consider, which could have superior performance. This includes other small sample corrections available for GEE [10, 25, 27, 52]; some of these could have improve the type-one error control of GEE particularly for scenarios with large variability in cluster size [25], an over-dispersed binomial model [53, 54], and non-parametric methods such as permutation tests. Our analysis methods have not adjusted for covariates, and our simulations used simple randomisation. The methods we have considered may all be impacted by adjustment for cluster-level covariates. We have only considered estimation of the intervention effect, which is a cluster-level covariate. The performance of GEE methods may have been impacted by comparison to estimated marginal effects because our data generating mechanism used cluster-specific effects. We excluded any runs that did not converge. This was very common for GEE, and may have biased the estimated power if runs were more or less likely to converge if they identified an intervention effect: to our knowledge, this was not the case. Further, we only considered prevalence as low an 10%.

## Conclusion

We recommend that CRTS with 30 or fewer clusters and a binary outcome use an unweighted cluster-level analysis, or GLMM using REPL. Confidence intervals and p-values for both methods should be calculated based on a t-distribution with the number of degrees of freedom defined as the number of clusters minus cluster-level parameters.

## Supplementary Information

**Additional file 1.** Supporting Information.

**Additional file 2. **Code to replicate illustrative example.

## Data Availability

Data for the illustrative example are available at http://doi.org/10.17037/DATA.4. Code to implement the methods discussed in this article is provided in the supplementary material.
