# Supplementary material for: Cluster randomised trials with a binary outcome and a small number of clusters: comparison of individual and cluster level analysis method
Source: BMC Med Res Methodol. 2022 Aug 12;22:222. doi: 10.1186/s12874-022-01699-2 (PMC9375419; doi:10.1186/s12874-022-01699-2)

Supporting Information

Supporting text

Cluster level weights derivation

From a Taylor series expension, the variance of the cluster log-odds is approximated by

$$var\left(log\left[\frac{\hat{p}_{ij}}{1-\hat{p}_{ij}}\right]\right) \approx \sigma_b^2 + E\left[\frac{1}{p_{ij}(1-p_{ij})m_{ij}}\right]$$

We can substitute $\rho_i = \frac{\sigma_b^2}{\sigma_b^2 + \frac{1}{p_i(1-p_i)}}$ and take the prevalance in each arm for the expectation of the prevlance in each cluster so that

$$var\left(log\left[\frac{\hat{p}_{ij}}{1-\hat{p}_{ij}}\right]\right) \approx \frac{\rho_i}{(1-\rho_i)p_i(1-p_i)} + \frac{1}{p_i(1-p_i)m_{ij}}$$

$$= \frac{1+(m_{ij}-1)\rho_i}{m_{ij}(1-\rho_i)p_i(1-p_i)}$$

Inverse variance weights are then

$$w_{ij} = \frac{m_{ij}(1-\rho_i)p_i(1-p_i)}{1+(m_{ij}-1)\rho_i}$$

Assuming the same mean prevalance in both arms and the same ICC in both arms, we can simplify this to

$$w_{ij} = \frac{m_{ij}}{1+(m_{ij}-1)\rho_i}$$

Data simulation: Distribution of random effects

We simulated scenarios with u_{ij} sampled from three different distributions.

Normal distribution: In this scenario, $u_{ij} \sim N(0, sigma_b)$

Gamma distribution: In this scenario, $u_{ij} = \frac{\sigma_b(a_{ij}-2)}{\sqrt{2}}$ and $a_{ij} \sim Gamma(2,1)$

The shape parameter of the Gamma disitribution was set to 2 as a way of comparing small sample performance to large sample performance with non-normal random effects. Heagerty and Kurland [1] found in large samples, generalised linear mixed models gave cluster-level covariate estimates with little bias with a shape parameter of 2 or larger, but smaller values lead to biased effect estimates. We assess if this limit is similar with a small number of clusters.

This process gives values of u_{ij} with mean zero and variance $\sigma_b 2$ A shape parameter of 2 give a distribution with skew 1.4 and kurtosis 3.

Uniform distribution: In this scenario, $u_{ij} \sim N(-\sqrt{3\sigma_b^2}, \sqrt{3\sigma_b^2})$. This give a distribution with mean zero, variance σ_b^2, zero skew, and kurtosis -6/5.

Data simulation: Cluster size sampling

When cluster size varied we sampled m_{ij} to have a minimum cluster size of 3. Each scenario set the mean cluster size m and coefficient of variation of cluster size $CV = S/m$ where S is the standard deviation of cluster size. We set $m_{ij} = 2 + \delta_{ij}$ where

$$\delta_{ij} \sim Negbin\left(no.offails = \frac{(m-2)^2}{s^2 - (m-2)}, prob.offail = \frac{m-2}{s^2}\right)$$

References

[1] HEAGERTY, P. J. & KURLAND, B. F. 2001. Misspecified maximum likelihood estimates and generalised linear mixed models. Biometrika, 88, 973-985.

Figure 1: Examples of cluster-level summary distrubtions where analysis methods have nominal type-one error in previous literature.

A: The t-test has been shown to gave close to nominal type-one error when observations follow an exponential distribution with rate = 1 even in small samples (Boneau 1960) B: GLMMs (large number of clusters) have been shown to give close to nominal type one error for fixed effects when cluster-means follow a gamma distribution with skewness $2/\sqrt{(2)}$. Type-one error is affected with more severe skew (Litiere 2008). C: Both t-tests (large and small samples) and random effect models (large number of clusters) have been shown to have nominal type-one error with a uniform distribution.

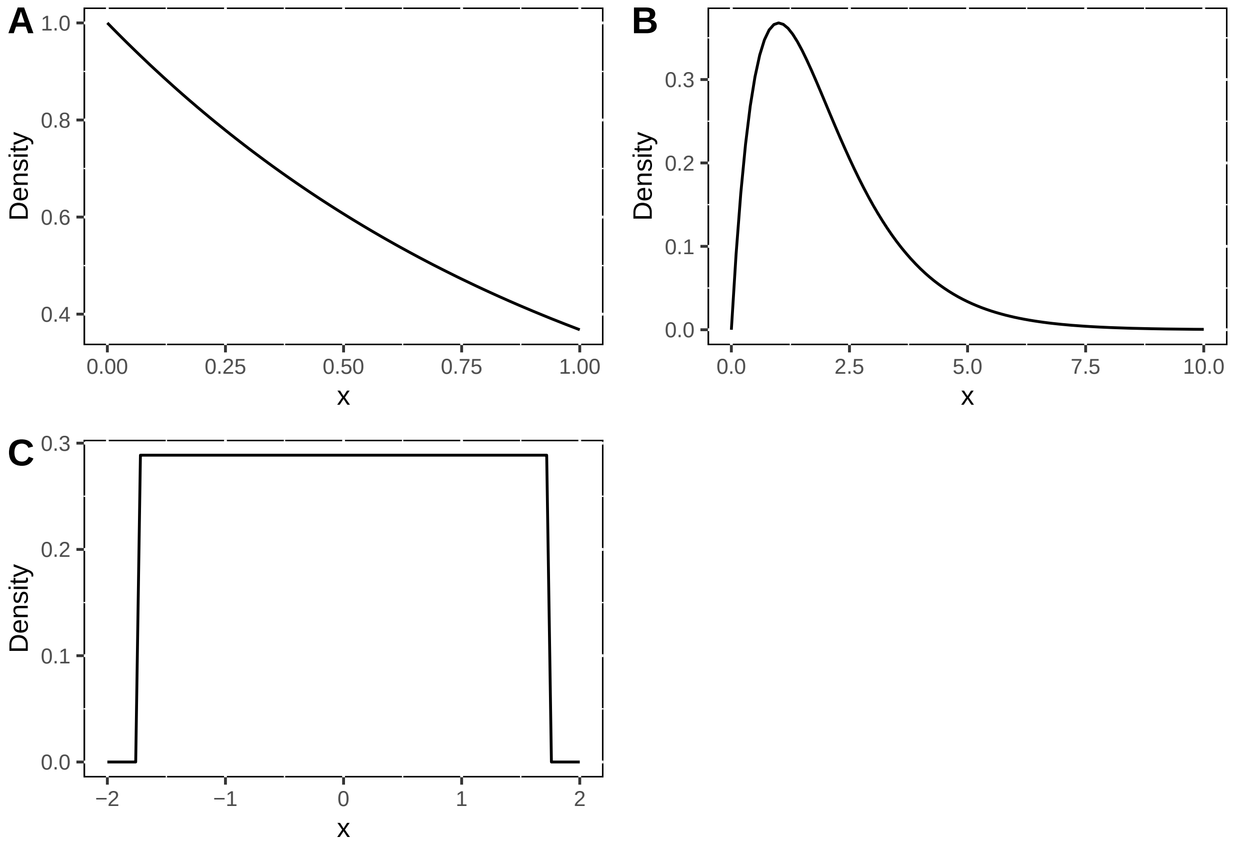

Figure 2: Performance measures of cluster-level analysis methods by number of clusters (rows) and prevalence (colour). Measures shown (columns): Standardised intervention effect estimate bias, Standard error bias, Type-one error

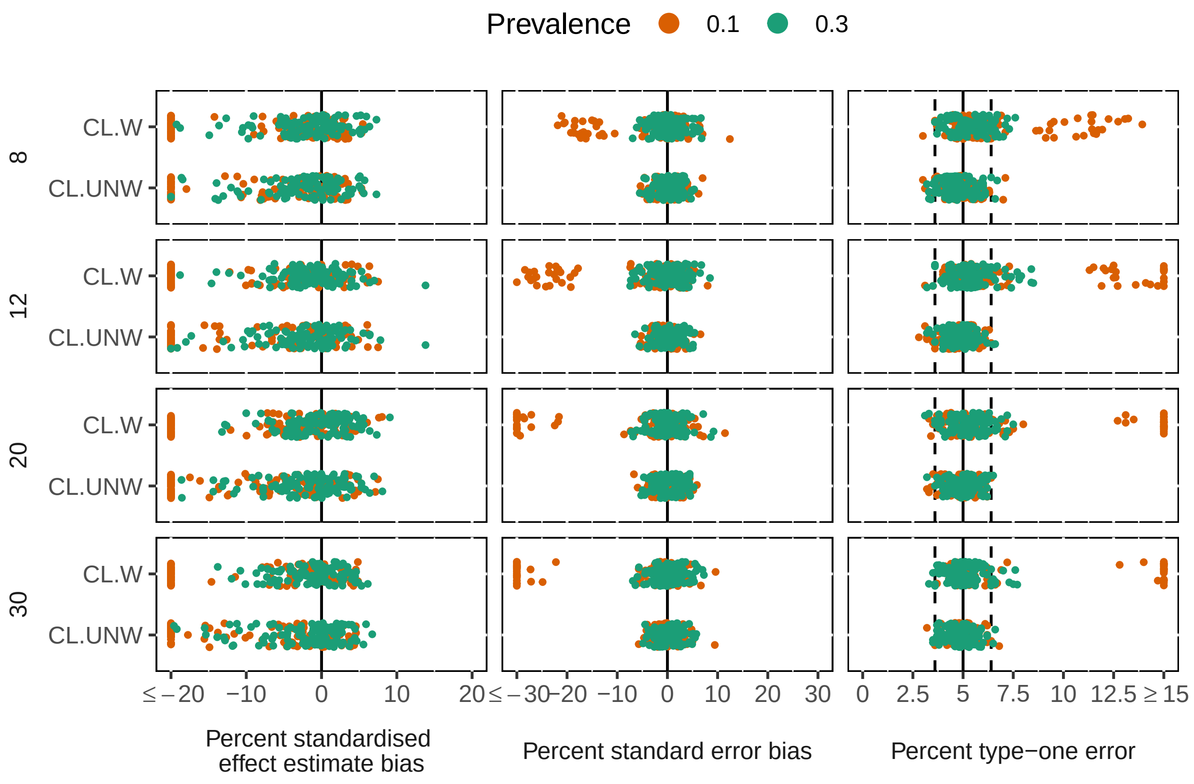

Figure 3: Performance measures of cluster-level analysis methods by number of clusters (rows) and distribution of cluster log-odds (colour). Measures shown (columns): Standardised intervention effect estimate bias, Standard error bias, Type-one error

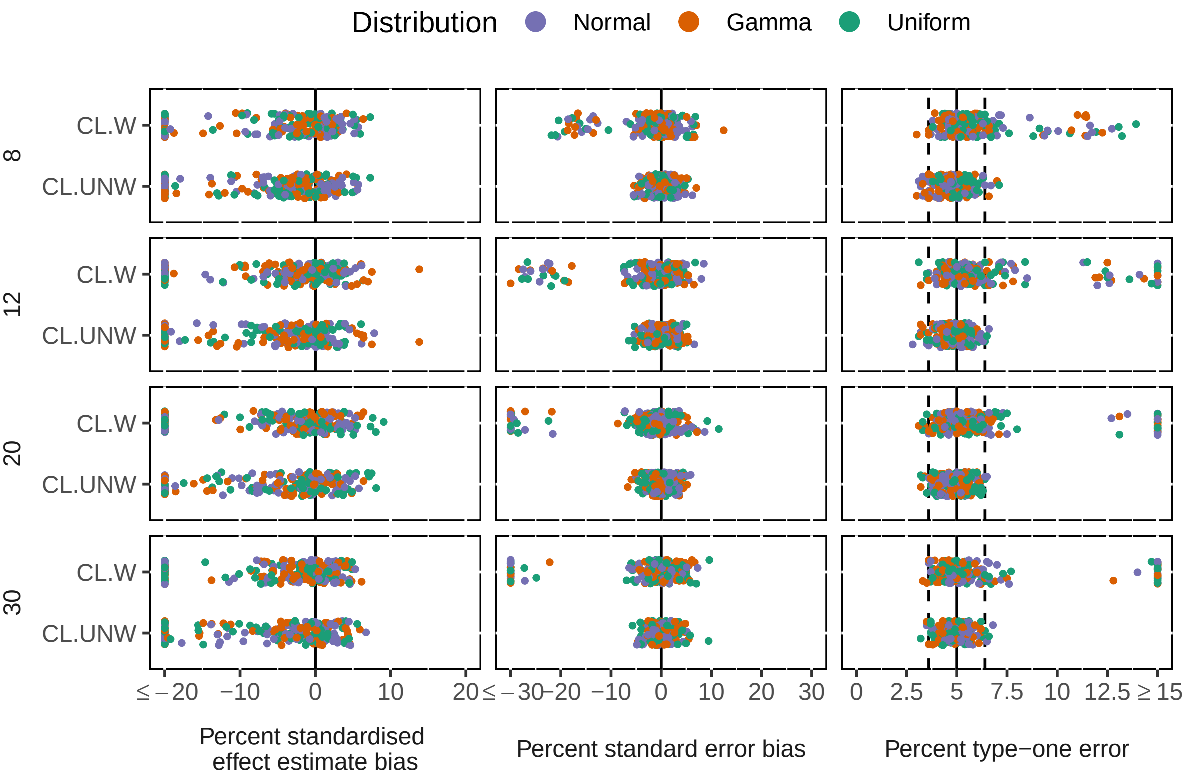

Figure 4: Performance measures of cluster-level analysis methods by number of clusters (rows) and mean cluster size (colour). Measures shown (columns): Standardised intervention effect estimate bias, Standard error bias, Type-one error

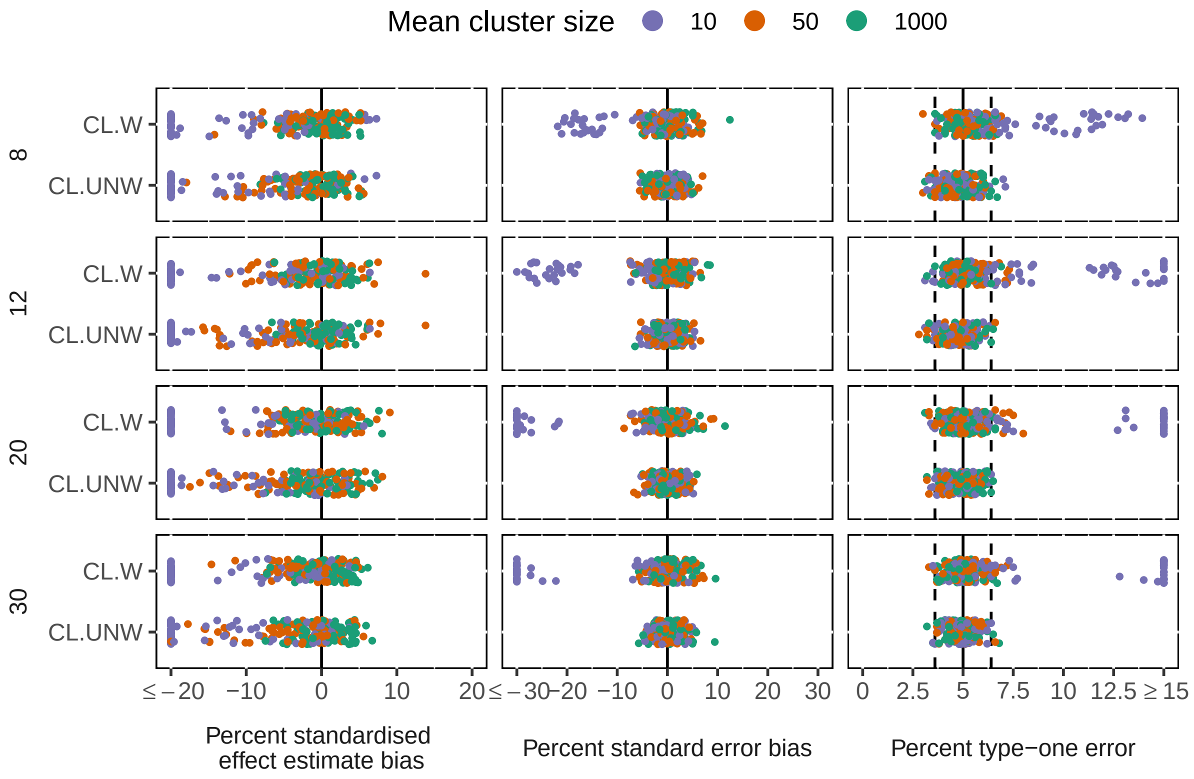

Figure 5: Performance measures of cluster-level analysis methods by number of clusters (rows) and CV of cluster size (colour). Measures shown (columns): Standardised intervention effect estimate bias, Standard error bias, Type-one error

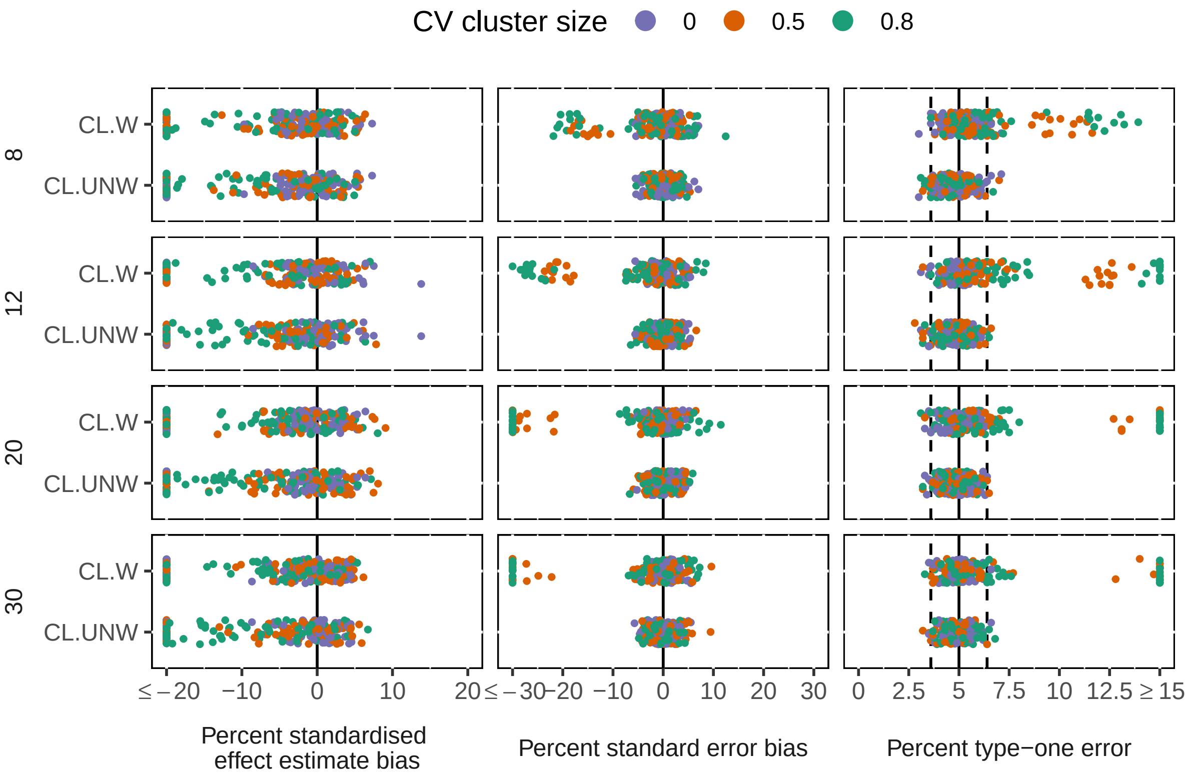

Figure 6: Performance measures of cluster-level analysis methods by number of clusters (rows) and ICC (colour). Measures shown (columns): Standardised intervention effect estimate bias, Standard error bias, Type-one error

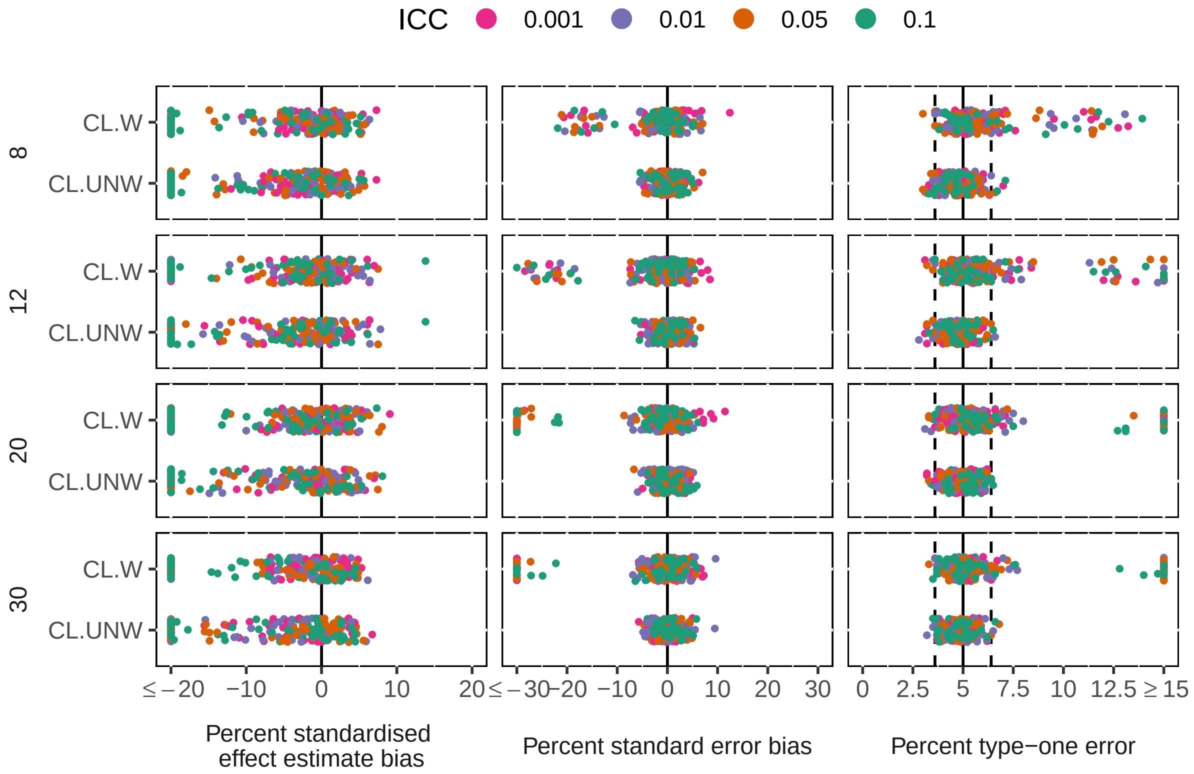

Figure 7: Performance measures of GLMM methods by number of clusters (rows) and prevalence (colour). Measures shown (columns): Effect estimate bias, Standard error bias, Type-one error

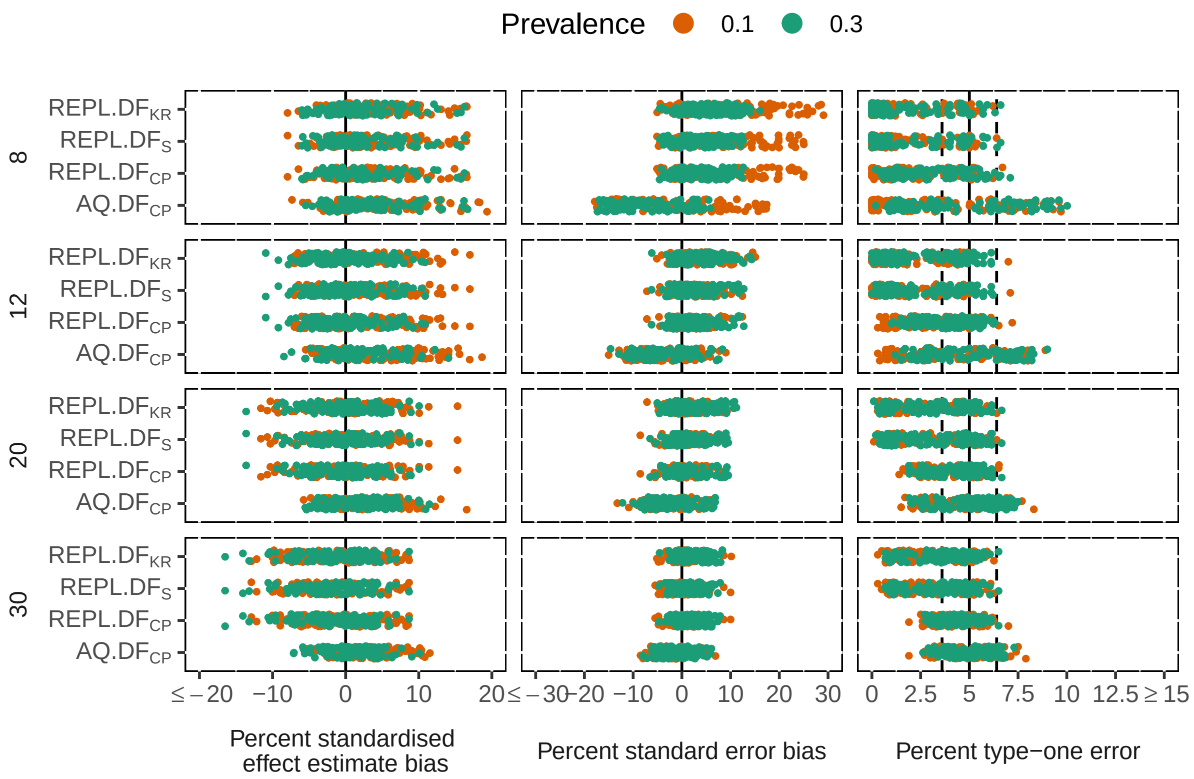

Figure 8: Performance measures of GLMM methods by number of clusters (rows) and distribution of cluster log-odds (colour). Measures shown (columns): Effect estimate bias, Standard error bias, Type-one error

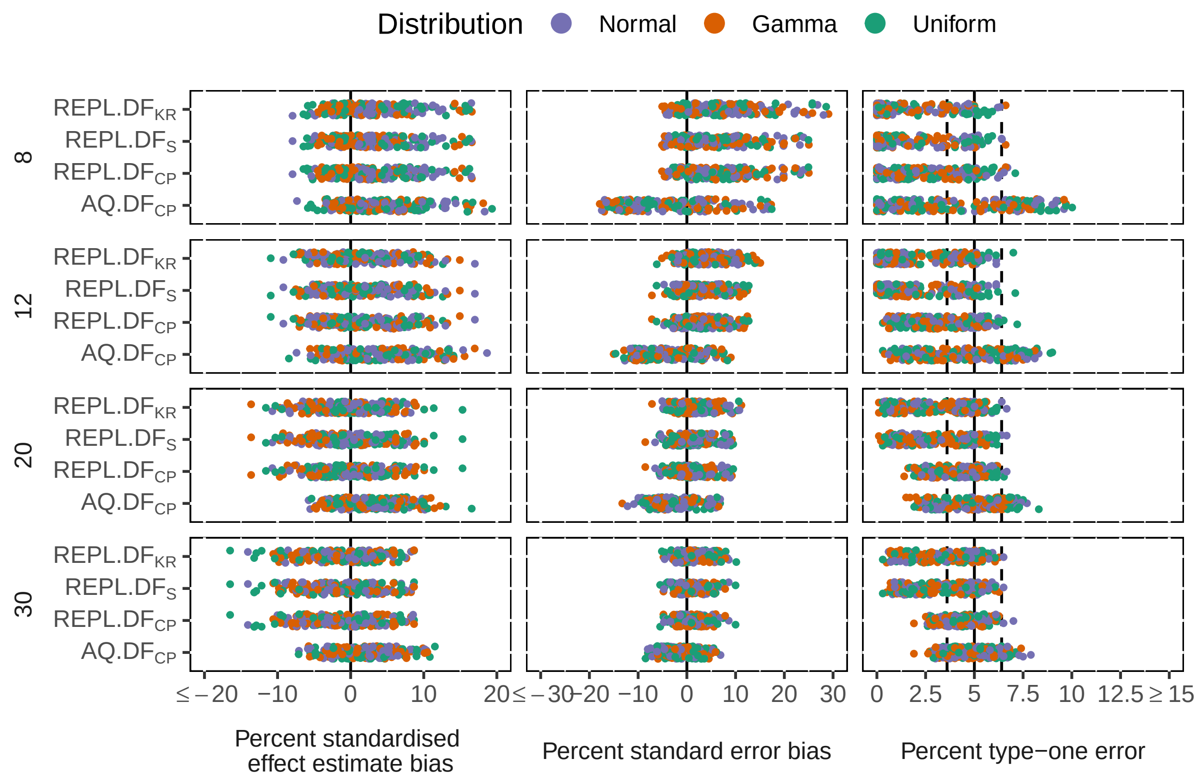

Figure 9: Performance measures of GLMM methods by number of clusters (rows) and CV of cluster size (colour). Measures shown (columns): Effect estimate bias, Standard error bias, Type-one error

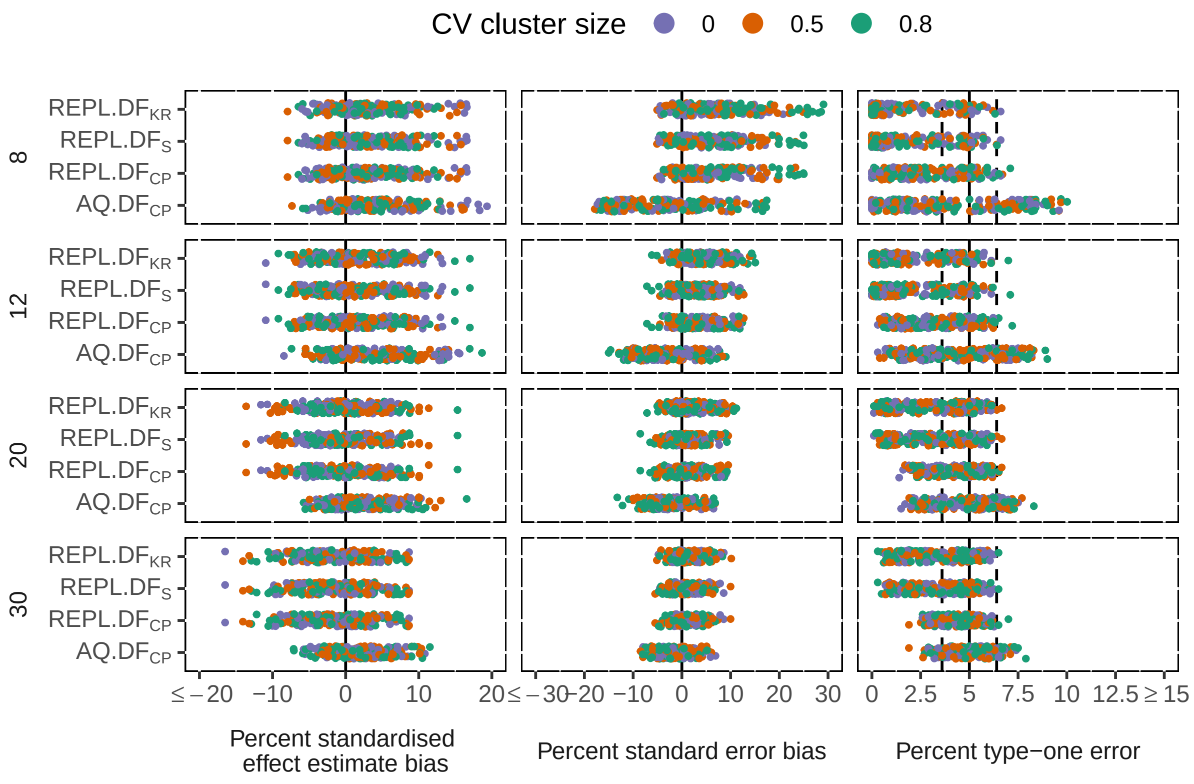

Figure 10: Performance measures of GLMM methods by number of clusters (rows) and ICC (colour). Measures shown (columns): Effect estimate bias, Standard error bias, Type-one error

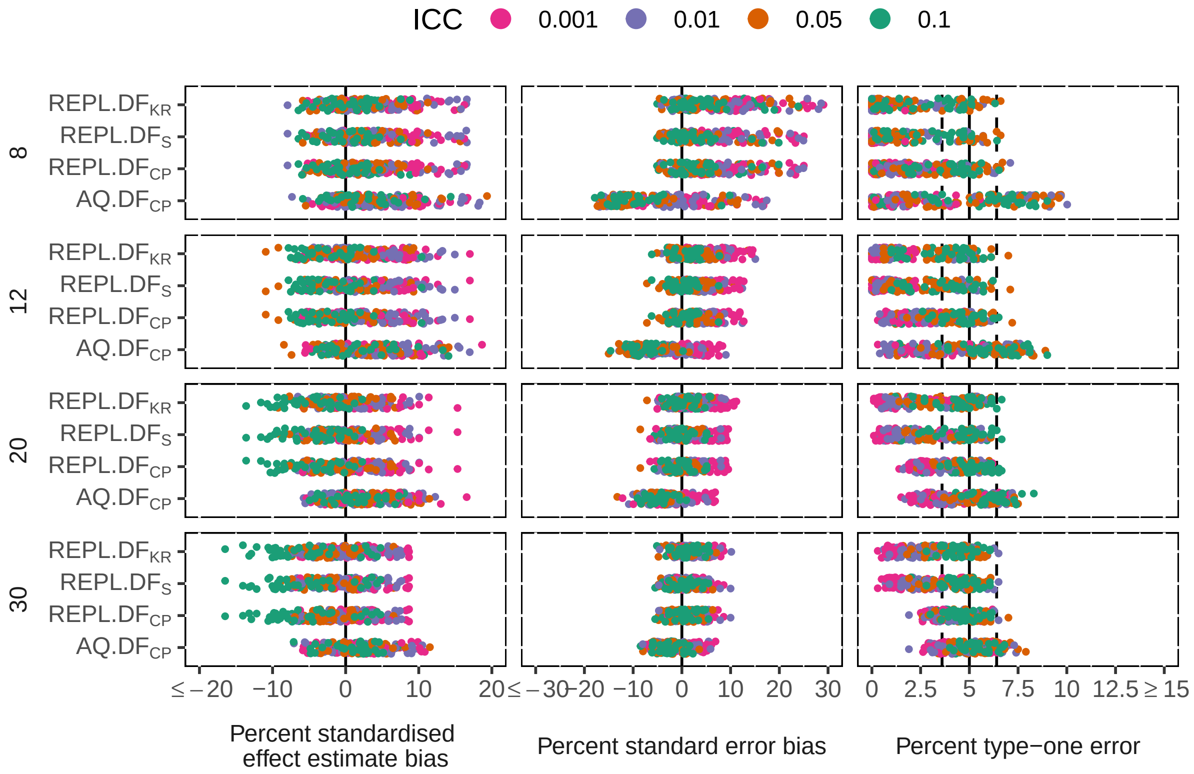

Figure 11: Comparison of bias and type-one error of GEE methods by number of clusters (rows) and prevalance (colour). Measures shown (columns): Effect estimate bias, Standard error bias, Type-one error.

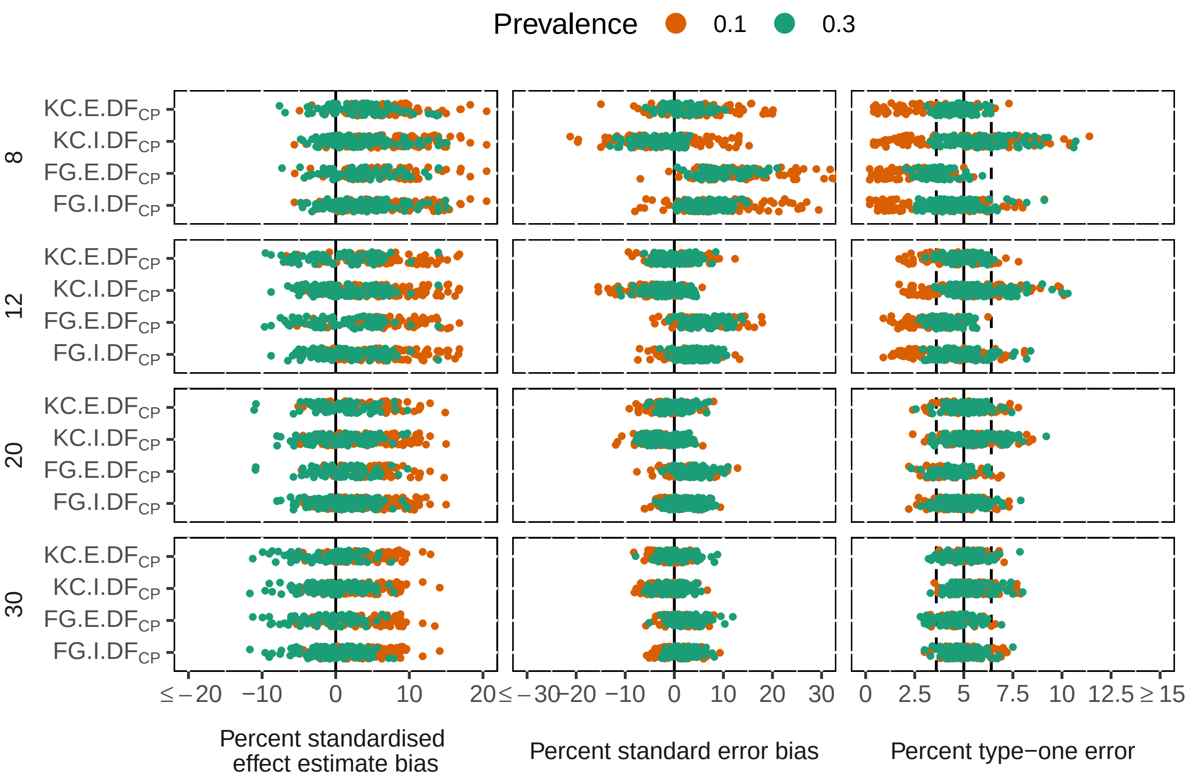

Figure 12: Comparison of bias and type-one error of GEE methods by number of clusters (rows) and distribution of cluster log-odds (colour). Measures shown (columns): Effect estimate bias, Standard error bias, Type-one error.

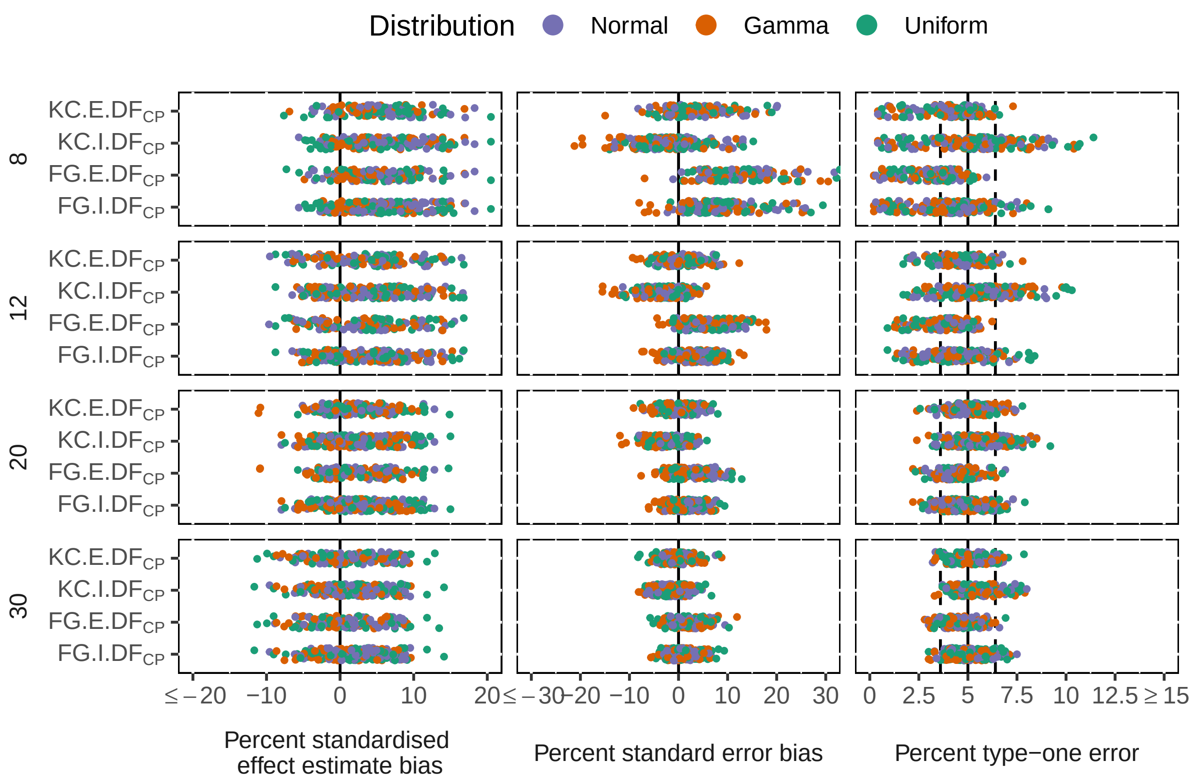

Figure 13: Comparison of bias and type-one error of GEE methods by number of clusters (rows) and CV of cluster size (colour). Measures shown (columns): Effect estimate bias, Standard error bias, Type-one error.

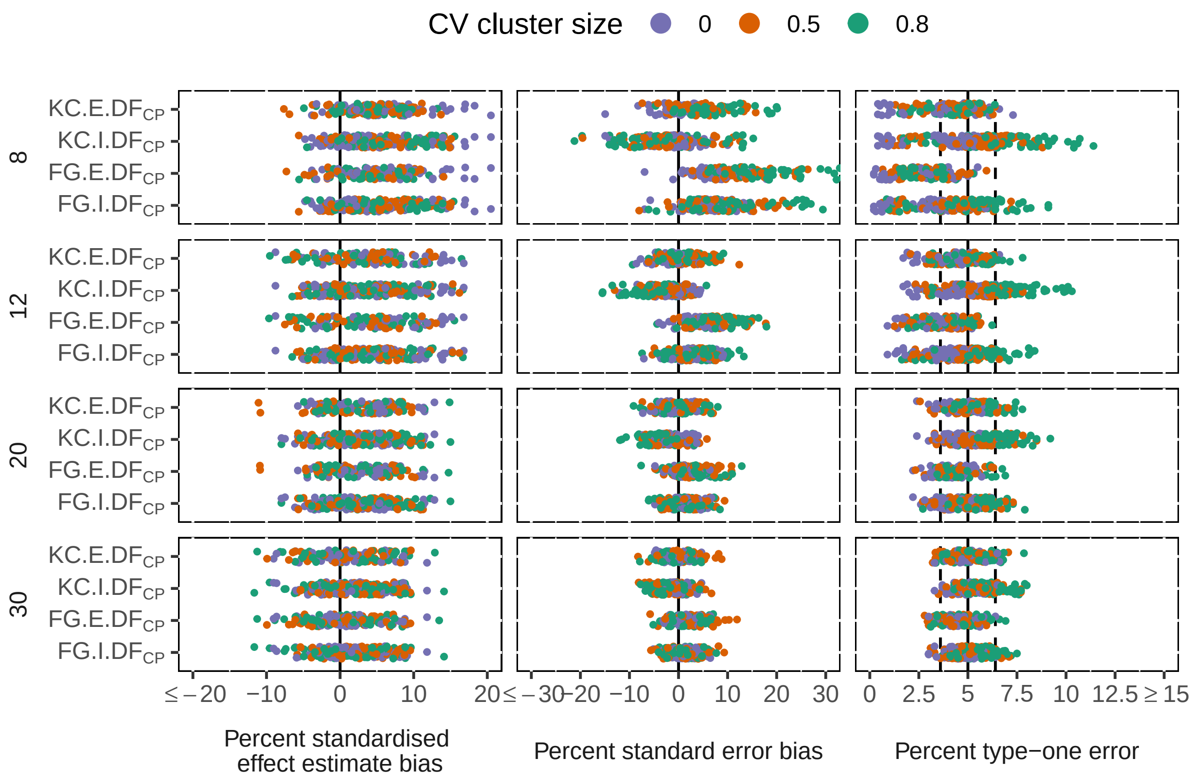

Figure 14: Comparison of bias and type-one error of GEE methods by number of clusters (rows) and ICC (colour). Measures shown (columns): Effect estimate bias, Standard error bias, Type-one error.

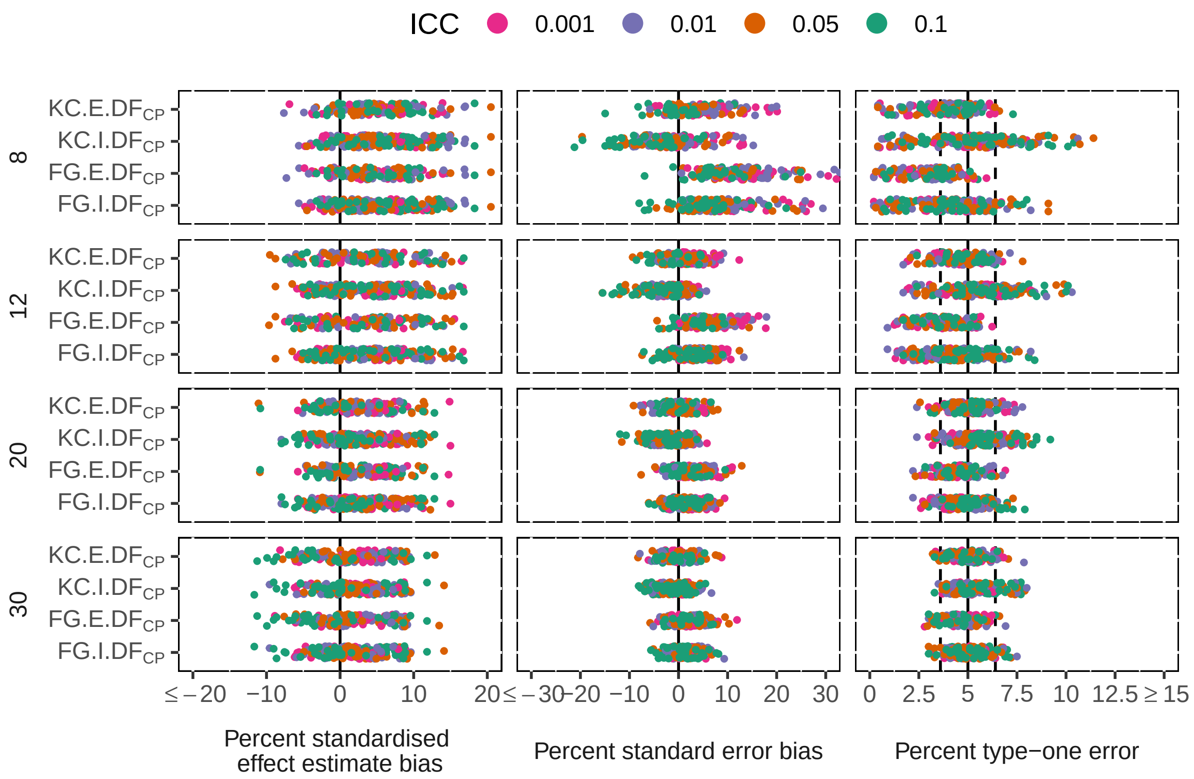

Figure 15: Power comparison of equally weight cluster level analysis, GLMM with REPL, and GEE with FG standard errors (columns) by number of clusters (rows), ICC (y axis), and prevalence (colour). All methods use $\mathbf{DF_{CP}}$

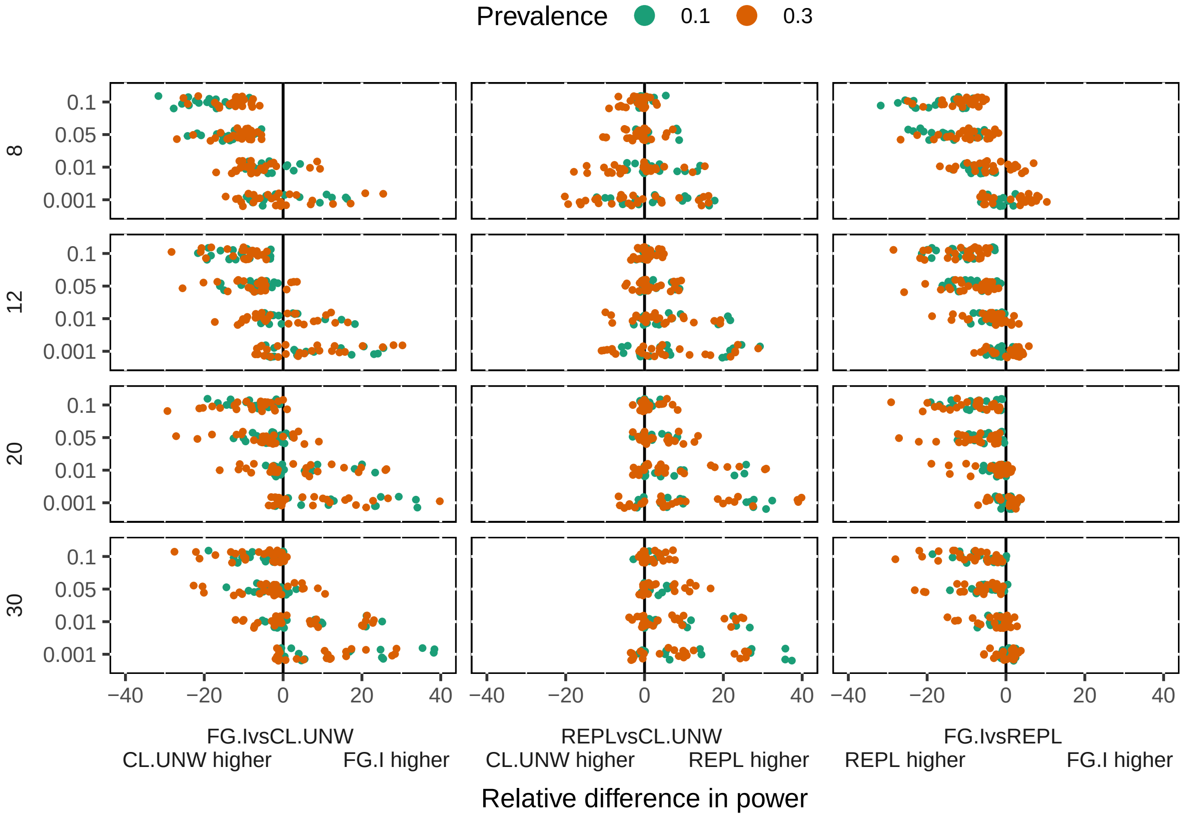

Figure 16: Power comparison of equally weight cluster level analysis, GLMM with REPL, and GEE with FG standard errors (columns) by number of clusters (rows), ICC (y axis), and distribution of cluster log-odds (colour). All methods use $\mathbf{DF_{CP}}$

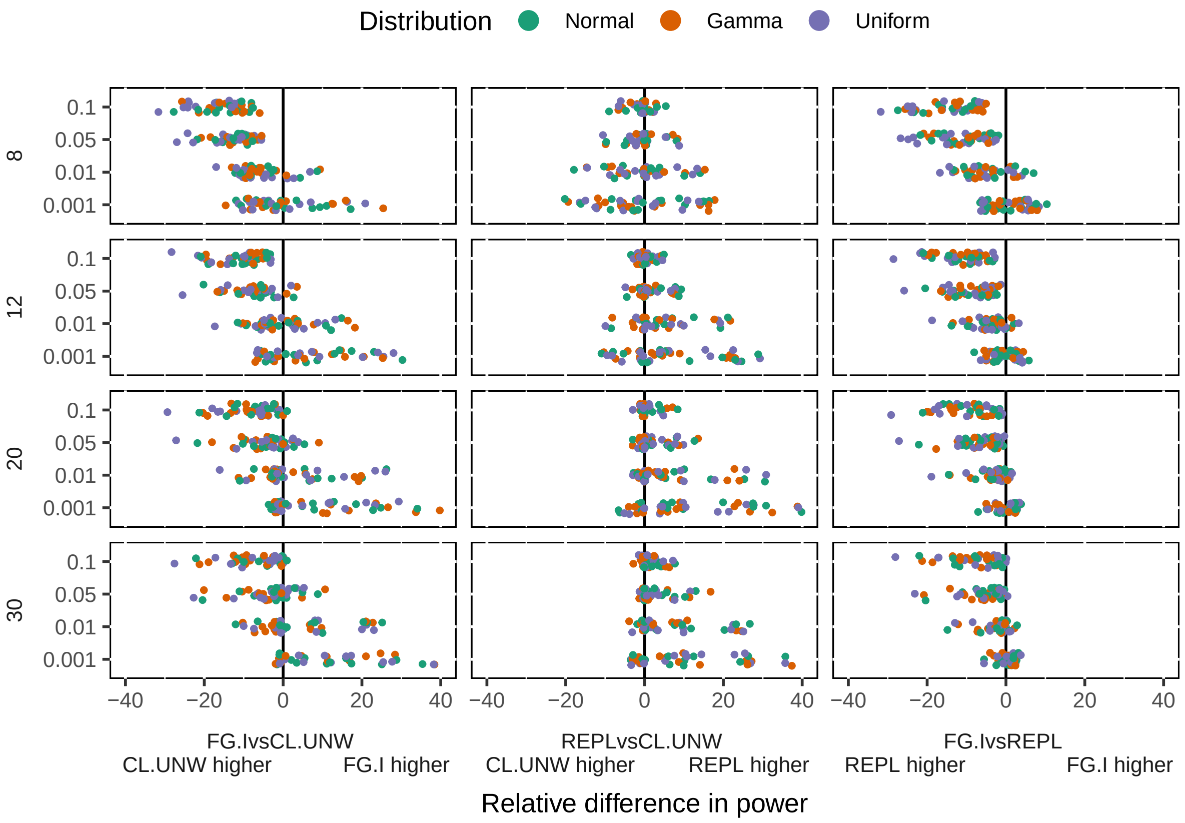

Figure 17: Power comparison of equally weight cluster level analysis, GLMM with REPL, and GEE with FG standard errors (columns) by number of clusters (rows), ICC (y axis), and mean cluster size (colour). All methods use $\mathrm{DF_{CP}}$

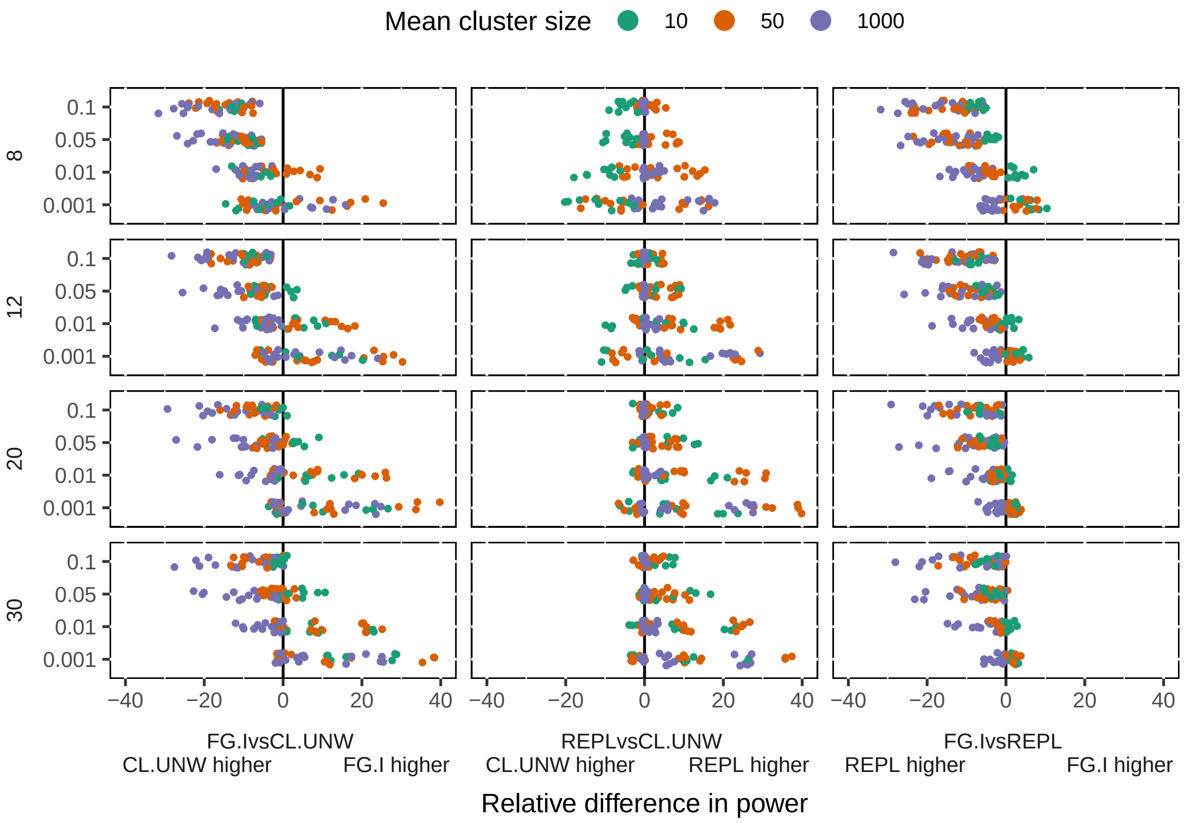

Supplement: Supplementary file 1 — Additional file 1. Supporting Information. [file 12874_2022_1699_MOESM1_ESM.pdf]
